# From acidification to multi-targeted cellular modulation: dual impact of hydroxy acids on the skin microbiota and the epidermal barrier

**DOI:** 10.3389/fcimb.2026.1918758

**Published:** 2026-09-11

**Authors:** Jakub Szewczyński, Małgorzata Mazur, Hanna Tomczak

**Affiliations:** 1 Faculty of Medicine and Health Sciences, University of Kalisz, Kalisz, Poland; 2 WSB University in Poznan, Poznan, Poland

**Keywords:** biofilms, *Cutibacterium acnes*, dysbiosis, glycolic acid, lactobionic acid, salicylic acid, skin microbiome, *Staphylococcus epidermidis*

## Abstract

Maintaining proper skin surface acidity is essential for both epidermal barrier integrity and microbial homeostasis. This review examines the dual impact of hydroxy acids on the host-microbiota interface, focusing on their antimicrobial mechanisms, inflammatory modulation, and barrier remodeling. Traditional agents, such as glycolic and salicylic acids, induce exfoliation through desmosomal disruption and exert bactericidal effects via intracellular acidification. Salicylic acid specifically penetrates pilosebaceous units to suppress sebocyte lipogenesis, attenuate pro-inflammatory cascades, and downregulate bacterial virulence factors. However, aggressive exfoliation can compromise the epidermal barrier, which increases the risk of secondary dysbiosis. In contrast, next-generation polyhydroxy acids (such as lactobionic acid) reinforce structural cohesion and inhibit matrix metalloproteinases. These newer agents also, as demonstrated by *in vitro* models, can disrupt the bacterial cell envelope, destabilize biofilms, and are hypothesized to potentially interact with microbial DNA without causing severe irritation. Future therapeutic approaches need to balance clinical macroscopic endpoints with the preservation of microbiome diversity to avoid chronic microenvironment impairment. Our findings demonstrate how acid-induced pH reduction directly modulates bacterial virulence factors and suggests a potential to modulate microbial environments.

## 1 Introduction

Facial skin is a unique ecological niche harboring a specialized microbiome. Constantly exposed to environmental factors like ultraviolet (UV) radiation and temperature fluctuations, colonizing microorganisms must develop robust adaptive mechanisms (Sun et al., 2024; Tao et al., 2024). The natural facial pH of ~4.7 optimizes commensal survival and proliferation (Lambers et al., 2006). However, this delicate ecosystem faces intense pressure from the widespread use of low-pH exfoliants. In dermatology, α-hydroxy acids (AHAs) and β-hydroxy acids (BHAs) are standard tools for epidermal modulation, acting by weakening corneocyte adhesion to accelerate exfoliation and renewal (Fartasch et al., 1997). While therapeutically valuable, aggressive “overexfoliation” compromises the epidermal barrier, leading to increased transepidermal water loss (TEWL) and altered lipid composition (Elias, 2005; Proksch et al., 2008). Despite its prevalence, the impact of routine exfoliation on skin microbiota remains incompletely understood (Callejon et al., 2023; Dubli et al., 2025). Consequently, there is a paradigm shift toward microbiome-sparing therapies, barrier-preserving hygiene, and conservative management (e.g., probiotics, diet), which yield substantial benefits for cutaneous homeostasis. Given that intensive hydroxy acid application can disrupt this homeostatic balance, a profound understanding of its microbial impact is essential. Therefore, this review systematically and critically evaluates the direct impact of first- and next-generation hydroxy acids on cutaneous microbiota and barrier integrity. By explicitly addressing the limitations of current *in vitro* models, it aims to bridge the gap between clinical topical applications and medical microbiology.

### 1.1 Characteristics of the face as a unique ecological niche

Cutaneous microbiota depends heavily on local microenvironments, including humidity, temperature, and stratum corneum (SC) thickness. Bacterial composition and density vary significantly across sebaceous, moist, or dry sites (Grice and Segre, 2011). Exposed areas under continuous environmental stress exhibit the lowest microbial burden, dominated by β-Proteobacteria and Flavobacteriales. Conversely, sebaceous regions harbor higher densities of lipophilic yeasts (*Malassezia* spp.) and bacteria. Propionibacterineae dominate the scalp, nose, ears, and hair follicles (Costello et al., 2009; Grice and Segre, 2011). Hormonal dimorphism also drives microbiome variations, while topical treatments significantly impact microbiota composition (Holland and Bojar, 2002). The highly sebaceous face is predominantly colonized by *Cutibacterium* (formerly *Propionibacterium*), alongside *Staphylococcus*, *Corynebacterium*, and *Malassezia*. The cheeks exhibit the highest diversity, representing the overall facial microbiome. Aging (>55 years) decreases *Cutibacterium acnes* and increases *Corynebacterium kroppenstedtii*, correlating with reduced collagen biosynthesis and elevated oxidative stress. Commensals like *Staphylococcus epidermidis* provide frontline defense, secreting antimicrobial peptides (AMPs) to inhibit opportunistic pathogens like *Staphylococcus aureus*. Additionally, air pollution, smoking, and preservative-containing topical formulations can disrupt the microbiome network and increase TEWL, altering biodiversity (Keum et al., 2020; Sfriso et al., 2020; Hwang et al., 2021; Lee et al., 2025, Lee et al., 2021; Russo et al., 2023; Garlet et al., 2024; Wagner et al., 2024; Hong et al., 2025). The homeostasis of cutaneous microflora remains vulnerable to both exogenous stressors, such as aggressive exfoliation and pollution, and systemic factors like antibiotic therapy. Chemical peels—primarily AHAs (e.g., glycolic acid [GA], lactic acid [LA]) and BHAs (e.g., salicylic acid [SA])—are widely used. While clinical concentrations (AHAs >10-15%, BHAs >2%) are strictly regulated, daily home use of sub-threshold formulas poses overutilization risks. Skin and microbiota health depend heavily on concentration, pH, and application frequency. To mitigate irritation, larger-molecular-weight polyhydroxy acids (PHAs, e.g., lactobionic acid [LBA]) with superior tolerability have emerged, alongside composite formulations enhancing moisture retention. Ultimately, chemical peels can transiently impair the barrier, elevating TEWL and profoundly impacting the microbiome (Rodan et al., 2016). **Table 1** synthesizes the physicochemical properties, host-modulating pathways, bactericidal activity, and safety profiles of AHAs, BHAs, and PHAs within the epidermal-microbiome axis. To further delineate these differences across distinct biological endpoints, **Table 2** provides a comprehensive comparative matrix of their targeted antimicrobial, antibiofilm, and *in vivo* ecological impacts, highlighting key experimental models and current limitations.

**Table 1:** Integrative assessment of cutaneous penetration, molecular mechanisms, antimicrobial efficacy, and potential adverse effects of representative AHA, BHA, and PHA compounds.

Acid	Skin penetration	Mechanism of action on skin	Bactericidal activity	Potential side effects
Glycolic acid	Rapid and deep penetration through the entire stratum corneum (Soleymani et al., 2018; Almeman, 2024); may reach the dermis at high concentrations (Soleymani et al., 2018; Măgerusan et al., 2023). Depth is concentration-, time-, and pH-dependent (Babilas et al., 2012; Al-Talib et al., 2017; Soleymani et al., 2018; Tang and Yang, 2018; Măgerusan et al., 2023).	Loosens corneocyte adhesion via calcium ion chelation and cathepsin D stimulation (Hachem et al., 2010; Kornhauser et al., 2010). Induces corneocyte apoptosis via caspase-3, -8, and -9 pathways and TRPV1 channel activation (Denda et al., 2010; Tang and Yang, 2018; Almeman, 2024). Stimulates fibroblast synthesis of collagen I, elastin, and GAGs. Modulates IL-1α and inhibits tyrosinase activity (Kim et al., 1998; Okano et al., 2003; Kornhauser et al., 2010; Narda et al., 2021).	Exhibits pH-dependent, rapid bactericidal activity against *Cutibacterium acnes*, significantly reducing bacterial counts within minutes at highly acidic pH (Takenaka et al., 2012). Penetrates bacterial lipid membranes in an undissociated state, followed by cytoplasmic dissociation. This leads to destructive intracellular acidification, ATP depletion, and subsequent cell death (Cotter and Hill, 2003; Ricke, 2003)	Erythema, burning sensation, irritation, xerosis, erosive blisters, crusting (Nikalji et al., 2012; Sharad, 2013; Almeman, 2024). Risk of post-inflammatory hyperpigmentation (PIH), UV hypersensitivity, and herpes simplex exacerbation (Sharad, 2013; Almeman, 2024). Rarely: scarring (Sharad, 2013; Gerber et al., 2014).
Salicylic acid	Highly lipophilic (Arif, 2015; Soleymani et al., 2018); rapidly and freely penetrates lipid barriers into pilosebaceous units and hair follicles (Arif, 2015; Al-Talib et al., 2017; Soleymani et al., 2018). Poor penetration in sebum-depleted areas (Soleymani et al., 2018).	Keratolytic and desmolytic activity (Arif, 2015; Lu et al., 2019; Măgerusan et al., 2023). Inhibits lipogenesis and sebocyte proliferation via AMPK/SREBP-1 and NFκB pathways (Lu et al., 2019). Exerts strong anti-inflammatory effects (Arif, 2015; Lu et al., 2019; Măgerusan et al., 2023; Zhou et al., 2023). Activates PPAR pathways regulating keratinization (Zhou et al., 2023).	Exerts bacteriostatic and fungicidal effects by downregulating the transcription of essential virulence factors, including alpha-hemolysin and host-binding proteins (Kornhauser et al., 2010). Impairs *Staphylococcus aureus* biofilm dispersal by interfering with the *agr* quorum-sensing system, thereby decreasing the production of extracellular proteases and surfactant molecules (Dotto et al., 2021).	Xerosis, desquamation, burning sensation, erythema (Arif, 2015; Al-Talib et al., 2017; Shao et al., 2023), and microbiome disruption (Wojciechowska and Dos Santos Szewczyk, 2025). Risk of salicylism (systemic toxicity) if applied to large surface areas (Madan and Levitt, 2014; Tabatabai et al., 2025).
Lactobionic acid	Shallow and slow penetration due to high molecular weight (Green et al., 2009; Măgerusan et al., 2023; Piątek-Gołda et al., 2025); acts primarily on the surface to fortify the epidermal barrier (Green et al., 2009; Hachem et al., 2010). Selectively acidifies the lower stratum corneum (Hachem et al., 2010; Tasić‐Kostov et al., 2019).	Potent humectant and antioxidant properties (Coroli et al., 2021; Sáez‐Orviz et al., 2022; Măgerusan et al., 2023). Inhibits MMP-9 metalloproteinases, preserving collagen (Green et al., 2009; Coroli et al., 2021). Prevents deep corneodesmosome degradation by reducing serine protease activity (Hachem et al., 2010). Stimulates ceramide-generating enzymes and desmoglein-3 expression (Hachem et al., 2010).	Disrupts the structural integrity of the bacterial cell wall and membrane, inducing lethal leakage of intracellular contents, including alkaline phosphatase, proteins, and nucleotides (Cao et al., 2019; Kang et al., 2020; Sáez‐Orviz et al., 2022). Hypothesized to potentially interact with bacterial genomic DNA via intercalation (based on *in vitro* molecular modeling and cell-free assays) and normal cellular functions (Cao et al., 2019; Kang et al., 2020). Suppresses biofilm formation by reducing the release of extracellular EPS and eDNA, alongside inhibiting metabolic and hemolytic activities (Kang et al., 2024, Kang et al., 2020).	Highly tolerable with a low complication profile (Green et al., 2009; Tasić‐Kostov et al., 2019; Măgerusan et al., 2023). Occasional mild pruritus, burning, or erythema in sensitive individuals (Măgerusan et al., 2023; Piątek-Gołda et al., 2025). No irritant contact dermatitis has been reported in the current literature.

AHA, alpha-hydroxy acid; AMPK, AMP-activated protein kinase; BHA, beta-hydroxy acid; eDNA, extracellular DNA; EPS, extracellular polymeric substances; GAGs, glycosaminoglycans; IL-1α, interleukin-1 alpha; MMP-9, matrix metalloproteinase-9; NF-κB, nuclear factor kappa B; PHA, polyhydroxy acid; PIH, post-inflammatory hyperpigmentation; PPAR, peroxisome proliferator-activated receptor; SREBP-1, sterol regulatory element-binding protein 1; TRPV1, transient receptor potential vanilloid 1.

The table provides a comprehensive comparison of skin penetration dynamics, host-modulating pathways (epidermal and dermal), and direct bactericidal activity against key skin pathogens. Additionally, it outlines the safety profiles and potential adverse effects associated with chronic or high-concentration exposure to each acid group within the epidermal-microbiome axis.

**Table 2:** Comparative analysis of microbiological and epidermal effects of key hydroxy acid representatives (GA, SA, and LBA).

Class and acid	Antimicrobial activity	Antibiofilm activity	Impact on skin microbiome	Experimental models	Main limitations
AHA: Glycolic Acid	It exhibits moderate, low pH-dependent bactericidal and growth-inhibitory effects *in vitro* against *C. acnes* (Takenaka et al., 2012; Tang and Yang, 2018). Undissociated acid molecules can passively penetrate bacterial cell membranes (Ricke, 2003). There is no direct evidence for universal *in vivo* bactericidal action against other epidermal pathogens.	It shows potential to reduce epidermal bacterial colony counts, which may indirectly limit biofilm formation (Takenaka et al., 2012). It lacks rigorous evidence for direct destruction of mature biofilm matrices, as observations are limited to single-species laboratory models.	Peeling applications (e.g., 35% pH 1.2) cause a transient decrease in overall propionibacteria load on the skin surface *in vivo* (Takenaka et al., 2012). It lacks long-term, longitudinal analyses determining microbiome ecological stability (Wojciechowska and Dos Santos Szewczyk, 2025). Rapid pH drop and exfoliation may disrupt the barrier and increase TEWL, posing a dysbiosis risk (Fartasch et al., 1997; Wojciechowska and Dos Santos Szewczyk, 2025).	Single-species *in vitro* bacterial suspensions and simplified agar diffusion tests (Takenaka et al., 2012). Reconstructed skin equivalents (e.g., TESTSKIN) (Denda et al., 2010) and short-term, small-scale *in vivo* clinical trials (e.g., split-face) (Babilas et al., 2012; Narda et al., 2021).	Risk of epidermal irritation and inflammation, including TRPV1 stimulation and IL-1α release (Okano et al., 2003; Denda et al., 2010; Tang and Yang, 2018). Rapid pH drop without neutralization damages the barrier and increases TEWL (Fartasch et al., 1997; Babilas et al., 2012; Tang and Yang, 2018). Fast, uncontrolled penetration occurs due to its low molecular weight of 76 Da (Tang and Yang, 2018; Almeman, 2024).
BHA: Salicylic Acid	It exhibits direct bacteriostatic and bactericidal action *in vitro* (Arif, 2015). Due to high lipophilicity, it actively and selectively penetrates sebum-rich pilosebaceous units. This directly targets the ecological niches of acne pathogens (Arif, 2015; Al-Talib et al., 2017; Soleymani et al., 2018).	It can disrupt *S. aureus* biofilms *in vitro*, but this mechanism relies on weakening the quorum sensing (*agr*) system. Under specific laboratory conditions, this can paradoxically stabilize and preserve the existing biofilm matrix (Fournière et al., 2020; Dotto et al., 2021).	Clinical application of 30% SSA demonstrates proven microbiome modification in acne patients. This manifests as a transient drop in α-diversity, a significant reduction in *Staphylococcus* spp. proportions, and a downward trend for *Cutibacterium* spp. (*Propionibacterium* sp.) (Bilal et al., 2023; Shao et al., 2023).	*In vitro* suspension cultures, single-species bacterial biofilms, and animal models like SKH-1 mice (Kornhauser et al., 2010; Arif, 2015). Clinical trials in AV patients using high-throughput 16S rRNA gene sequencing (Bilal et al., 2023; Shao et al., 2023).	Risk of irritation, erythema, xerosis, and intense epidermal desquamation (Arif, 2015; Al-Talib et al., 2017; Soleymani et al., 2018). Risk of systemic salicylism if applied over large areas (Arif, 2015; Tabatabai et al., 2025). Paradoxical stabilization of certain biofilms *in vitro* by inhibiting the *agr* system (Fournière et al., 2020; Dotto et al., 2021).
PHA: Lactobionic Acid	*In vitro* antimicrobial activity occurs through cell envelope disruption, morphological deformations, AKP leakage, and hypothesized genomic DNA intercalation (observed exclusively in laboratory settings) (Kang et al., 2020; Sáez‐Orviz et al., 2022). There is no clinical evidence for identical bactericidal effects on skin *in vivo*.	Laboratory models show its ability to disrupt biofilm architecture by inhibiting eDNA and EPS release (Sáez‐Orviz et al., 2022; Kang et al., 2024). It is hypothesized to participate in regulating the *GlpQ* gene, which degrades cell wall teichoic acids (WTA) (Kang et al., 2024).	It exhibits strong *in vitro* bactericidal activity, such as against commensal *S. epidermidis* at concentrations >0.7 mg/mL (Coroli et al., 2021). However, there is a complete lack of *in vivo* clinical studies evaluating the impact of LBA (e.g., 10%) on natural facial microbiome ecology and diversity.	Almost exclusively single-species, simplified *in vitro* laboratory models in liquid media (Cao et al., 2019; Kang et al., 2020; Coroli et al., 2021; Kang et al., 2024). Often uses non-resident facial skin strains, such as *Pseudomonas fluorescens*, *Vibrio parahaemolyticus*, and *Salmonella* spp. (Kang et al., 2020; Sáez‐Orviz et al., 2022).	Extreme molecule hydrophilicity limits penetration into lipophilic pilosebaceous units (Tasić‐Kostov et al., 2019; Coroli et al., 2021). This hinders direct access to niches inhabited by *C. acnes* and *Malassezia* spp. Extrapolating test-tube MIC/MBC data to the living skin ecosystem is a methodological error.

AHA, alpha-hydroxy acid; AKP, alkaline phosphatase; AV, acne vulgaris; BHA, beta-hydroxy acid; *C. acnes*, *Cutibacterium acnes*; eDNA, extracellular DNA; EPS, extracellular polymeric substances; GA, glycolic acid; LBA, lactobionic acid; MBC, minimum bactericidal concentration; MIC, minimum inhibitory concentration; PHA, polyhydroxy acid; *S. aureus*, *Staphylococcus aureus*; *S. epidermidis*, *Staphylococcus epidermidis*; SA, salicylic acid; SSA, supramolecular salicylic acid; TEWL, transepidermal water loss; TRPV1, transient receptor potential vanilloid 1; WTA, wall teichoic acids.

The table provides a comprehensive comparison of key hydroxy acids, explicitly distinguishing between their direct antimicrobial and antibiofilm activities, and their broader impact on the skin microbiome. Furthermore, the underlying experimental models and main limitations of current evidence are summarized.

## 2 Alpha-hydroxy acids

### 2.1 Physicochemical characteristics of α-hydroxy acids

AHAs are highly water-soluble weak acids characterized by an α-position hydroxyl group, exhibiting primarily superficial penetration. GA (pKa=3.83, 76 Da) is the most prevalent and extensively studied AHA (Green et al., 2009; Denda et al., 2010; Narda et al., 2021). Other commonly utilized AHAs in clinical dermatology include LA (pKa=3.86, 90 Da) and the aromatic, water, and organic solvent-soluble mandelic acid (MA; pKa=3.41, 152 Da) (Babilas et al., 2012; Soleymani et al., 2018; Măgerusan et al., 2023). With minimal molecular weight and size, GA possesses the greatest cutaneous penetration capacity; at high concentrations (70%), it acts as a medium-depth peel that penetrates the entire epidermis, reaching the papillary dermis (Soleymani et al., 2018; Măgerusan et al., 2023).

### 2.2 Mechanism of action and physiology of α-hydroxy acids

A widely proposed mechanism for AHA-induced exfoliation primarily relies on calcium ion chelation (Wang, 1999). Divalent Ca^2+^ ions are hypothesized to be essential for maintaining the cohesive integrity of desmosomal junctions. The chelation and subsequent removal of epidermal calcium ions is proposed to disrupt calcium-dependent cell adhesion molecules and initiate desquamation (Wang, 1999; Kornhauser et al., 2010), which manifests morphologically as a targeted, localized breakdown of corneosomes restricted to the stratum disjunctum (SD) (Fartasch et al., 1997). Topically applied AHAs are suggested to promote the dissociation of serine proteases from their endogenous inhibitor, LEKTI (lympho-epithelial Kazal-type-related inhibitor), through SC acidification (Deraison et al., 2007; Hachem et al., 2010). Microbiologically, maintaining the mildly acidic natural skin pH (~4.7) promotes commensals like *S. epidermidis* while hindering pathogens like *S. aureus* (Lambers et al., 2006; Choi and Kang, 2024). Although clinical studies evaluating facial microbiota α- and β-diversity shifts post-AHA application remain scarce, AHAs —most notably GA and MA—have been shown to exhibit growth-inhibitory or antibacterial activity against *C. acnes in vitro* (Takenaka et al., 2012; Măgerusan et al., 2023; Almeman, 2024). This direct antimicrobial effect, alongside the desquamatory and pH-regulating properties of other AHAs (such as LA, MA, and citric acid [CA]), collectively contributes to their clinical efficacy in managing acne (Babilas et al., 2012; Măgerusan et al., 2023; Almeman, 2024). However, extrapolating these *in vitro* susceptibility thresholds to firmly deduce direct modulation of the skin microbiome *in vivo* requires caution, as the complex cutaneous microenvironment and microbial resilience may significantly buffer these effects.

### 2.3 Glycolic acid

When analyzing the mechanism of action of GA, a fundamental physicochemical principle must be highlighted: the cutaneous efficacy of GA is contingent upon the pH and concentration of the applied formulation, as well as the duration of exposure (Babilas et al., 2012; Al-Talib et al., 2017). Consequently, a precise stratification of its clinical profile is imperative.

#### 2.3.1 Low-concentration and buffered formulations

**Figure 1:**
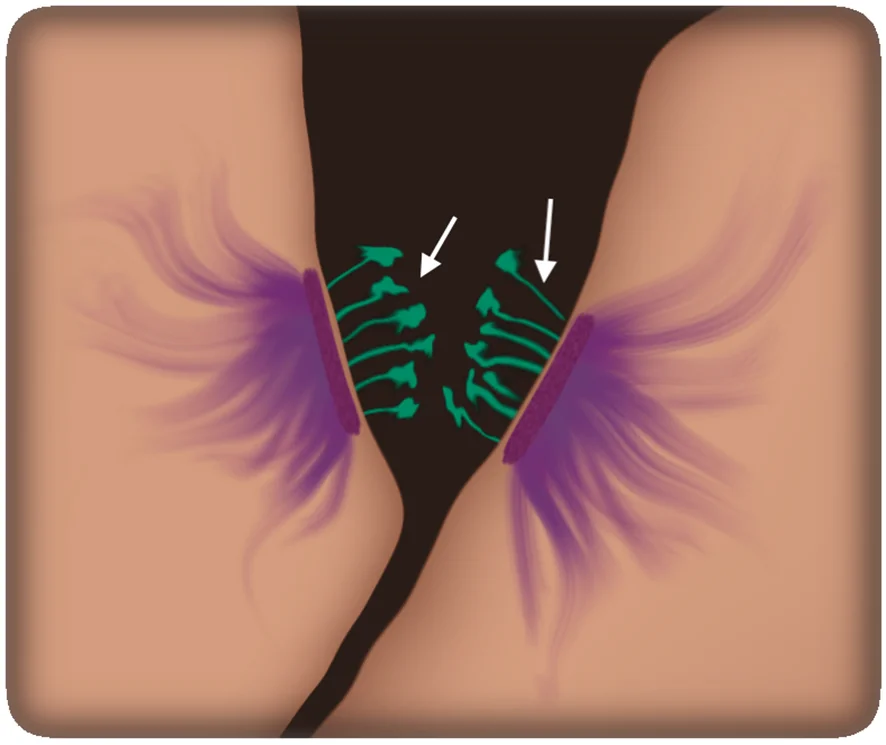
Modulatory effect of glycolic acid (GA) on epidermal cellular adhesions. The schematic depicts the controlled loosening of desmosomal structures (purple and green elements) between adjoining keratinocytes, facilitating physiological exfoliation. White arrows indicate the stretched and loosened desmosomal connections.*GA*, glycolic acid.

The impact of pH-optimized solutions relies on tissue stimulation without compromising the epidermal barrier (Fartasch et al., 1997; Narda et al., 2021). The utilization of GA-based formulations finds its specific role in skin regeneration, particularly in the context of photoaging (Babilas et al., 2012; Almeman, 2024). As demonstrated in HaCaT keratinocyte cell cultures, GA buffered to a pH of 7.1 at higher concentrations (5 mM) exhibits phototoxic and pro-apoptotic effects when combined with UVB radiation, whereas photoprotective activity was observed *in vitro* exclusively at lower concentrations (0.1 mM, pH 7.4) (Tang and Yang, 2018). Furthermore, *ex vivo* human skin models indicate that the application of formulations with a concentration ranging from 8% to 25% at a constant pH of 4.0 for 5 days also exerts a beneficial effect on keratinocyte proliferation and noticeably increases the thickness of the SC—owing to a looser assembly of corneocytes during desquamation—and collagen production (Narda et al., 2021). This GA-induced proliferative response is mediated by the activation of transient receptor potential vanilloid 1 (TRPV1) receptors, which triggers a rapid transient release of extracellular adenosine 5’-triphosphate (eATP) from epidermal keratinocytes in skin equivalent models (Denda et al., 2010). Parallel to these host-derived responses, GA-induced extracellular acidification may influence the cutaneous microbiota (Albert and Brown, 2015). The acid tolerance of *S. epidermidis* is hypothesized to be linked to its bioenergetic stability (Albert and Brown, 2015). While rapid extracellular acidification typically causes an immediate, proton-gradient-driven increase in cellular adenosine 5’-triphosphate (ATP) levels in other neutrophilic bacteria (such as *Escherichia coli* or *Bacillus subtilis*), *S. epidermidis* exhibits exceptional bioenergetic stability, showing variation in its cellular ATP levels under acidic conditions (Albert and Brown, 2015). This bioenergetic stability is suggested to provide an ecological advantage, enabling the commensal to maintain its metabolic homeostasis in low-pH environments induced by AHAs (Albert and Brown, 2015). The application of GA at lower concentrations (e.g., 4% to 10%) and a pH of 3.8 is clinically aimed at minimizing adverse effects (Fartasch et al., 1997; Babilas et al., 2012). Importantly, even under these mild conditions (such as 4% GA at pH 3.8), which preserve the epidermal barrier function, GA still accelerates desmosome degradation (**Figure 1**), acting within the SD (Fartasch et al., 1997).

GA is widely proposed as a chelating agent that reduces local calcium ion concentration, a mechanism known to disrupt epidermal cellular adhesions (Wang, 1999; Kornhauser et al., 2010). From a microbiological perspective, understanding GA’s direct impact on the cutaneous microbiota is critical. In an *in vitro* time-kill assay utilizing 30% GA buffered to a pH of 5.5, the survival of *C. acnes* was demonstrated for over 4 hours post-exposure initiation (Takenaka et al., 2012). Extrapolating the results from this isolated *in vitro* assay to the highly heterogeneous clinical environment is severely limited. Standard dermatological protocols for high-concentration GA peels typically involve short contact times (e.g., 2–5 minutes) prior to neutralization (Sharad, 2013), making a prolonged 4-hour exposure biologically unrepresentative. Furthermore, this planktonic assay does not account for the protective biofilm matrix and the complex microenvironment of *C. acnes* residing deep within the pilosebaceous unit (Fournière et al., 2020). Importantly, from an immunological and structural standpoint, this biochemical modulation by GA is highly precise. Human *in vivo* studies have demonstrated that the application of a 10% oil-in-water (O/W) GA emulsion (pH 4.0) induces a significant reduction in skin surface pH from an average of 5.2 to 4.0 (Babilas et al., 2012). Such acidification triggers the dissociation of kallikrein (KLK) enzymes from the inhibitory LEKTI complex (Deraison et al., 2007). The subsequent release of these active proteases further amplifies the desquamation process (Deraison et al., 2007). Concurrently, experimental models, including both *in vitro* cell cultures and *ex vivo* human skin explants, suggest that keratinocyte proliferation and SC thickening occur without inducing significant inflammation or stimulating the release of tumor necrosis factor-alpha (TNF-α) under pH-optimized or non-phototoxic concentrations (Denda et al., 2010; Tang and Yang, 2018; Narda et al., 2021). However, such formulations do trigger the release of interleukin-1α (IL-1α), which, as extrapolated from *in vitro* co-culture models and *ex vivo* tissue staining, can influence deeper cutaneous layers and stimulate dermal fibroblasts to produce matrix metalloproteinase-1 (MMP-1) and synthesize new collagen (Okano et al., 2003). From a microbiological perspective, although this GA-induced acidification theoretically creates an optimal microenvironment for the proliferation of the commensal *S. epidermidis* (Lambers et al., 2006; Choi and Kang, 2024), the acid itself concurrently exerts a concentration-dependent bactericidal effect, significantly inhibiting the growth of this beneficial bacterium at higher concentrations in *in vitro* assays (Lukic et al., 2021).

#### 2.3.2 High-concentration and acidic formulations

**Figure 2:**
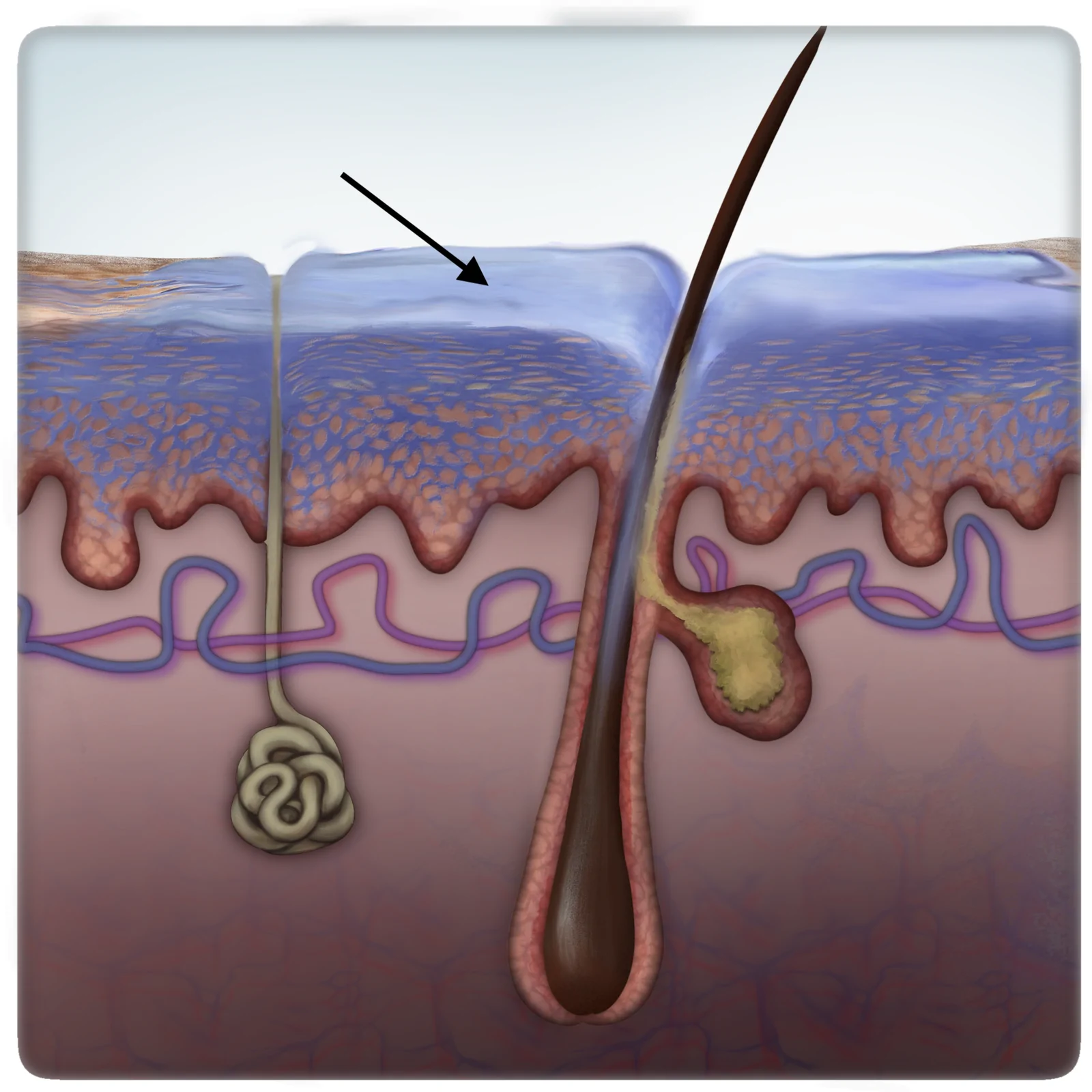
Cutaneous penetration profile of glycolic acid (GA). The uniform blue overlay illustrates the ability of this hydrophilic alpha-hydroxy acid to readily diffuse throughout the aqueous environment of the stratum corneum, ensuring superficial but widespread exfoliation. The black arrow denotes the superficial diffusion of the acid strictly within the stratum corneum.*GA*, glycolic acid.

The paradigm shifts drastically upon exceeding physiological tolerance barriers. Low-molecular-weight AHAs with the capacity for intensive penetration (**Figure 2**), such as GA (>70%), act as potent stressors on the skin (Nikalji et al., 2012; Sharad, 2013; Măgerusan et al., 2023). Clinical data and case reports have demonstrated that AHA formulations in concentrations ranging from 20% to 70% frequently exhibit a very low pH (<2) and possess a propensity to induce significant irritation and inflammation *in vivo* (Babilas et al., 2012; Gerber et al., 2014). According to research conducted *in vitro* on reconstructed human skin equivalent models, the application of low-pH (2.4) GA at high concentrations ranging from 1 M to 5 M for approximately 3 minutes, followed by washing and neutralization, induces a profound cellular stress response (Denda et al., 2010). This manifests as the extracellular release of ATP as a consequence of the activation of acid-sensing TRPV1 ion channels (Denda et al., 2010). Furthermore, *in vitro* studies on keratinocyte cell lines confirm that high concentrations of GA synergistically increase reactive oxygen species (ROS) and cytotoxicity (Tang and Yang, 2018). Consequently, in dermatological practice, GA at concentrations of 30–50% can elicit erythema, post-inflammatory hyperpigmentation, pruritus, or superficial epidermolysis in patients (Al-Talib et al., 2017; Soleymani et al., 2018; Almeman, 2024).

**Figure 3:**
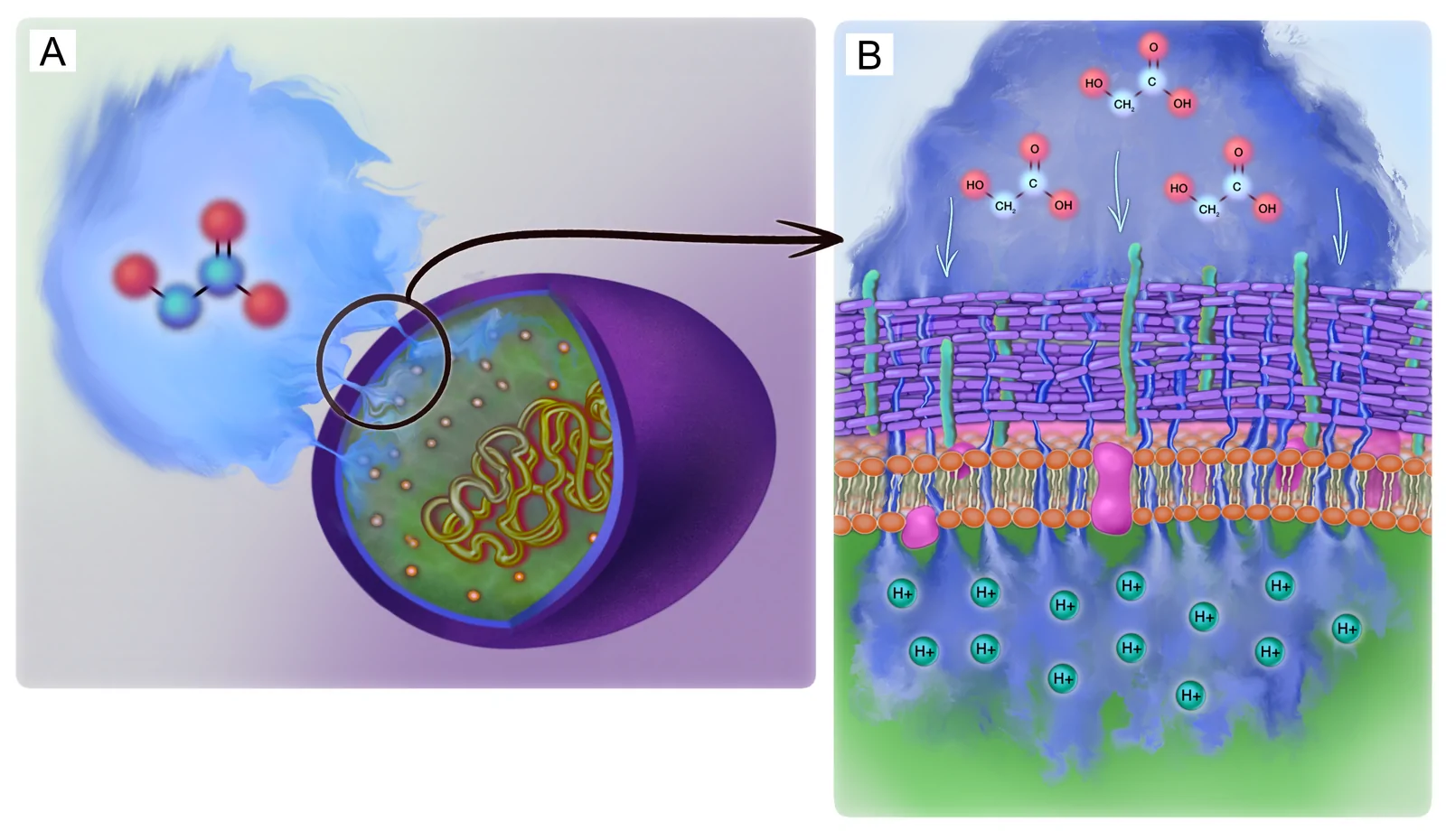
Conceptual model based on current literature illustrating the mechanism of GA-induced intracellular acidification in Gram-positive bacteria. **(A)** Glycolic acid **(GA)** approaches the bacterial cell envelope. **(B)** The black arrow pointing from panel **(B, A)** shows glycolic acid molecules diffusing across the cell wall and lipid bilayer. Light blue arrows indicate that GA molecules release hydrogen protons (H^+^), causing a rapid drop in intracellular pH. *GA*, glycolic acid; *H^+^*, hydrogen proton.

This aggressive exfoliation can also profoundly alter the composition and survival of the cutaneous microbiome (Shao et al., 2023; Wojciechowska and Dos Santos Szewczyk, 2025). In *in vitro* time-kill assays, the application of 30% GA at pH 1.5 and 3.5 for 5 minutes resulted in a reduction of the *C. acnes* (formerly classified as *Propionibacterium acnes*) population to less than 100 CFU/mL (Colony Forming Units) (Takenaka et al., 2012). *In vivo* studies utilizing a 35% GA formulation at pH 1.2 applied for approximately 2 minutes demonstrated a 94.2% reduction in surface *C. acnes* colony counts, compared to 54.2% in the control group treated solely with soap (Takenaka et al., 2012). The theoretical mechanism of the bactericidal activity of glycolic acid is illustrated in **Figures 3A, B**. However, a critical evaluation of these findings is necessary. While such extreme acidity (pH 1.2–1.5) exhibits undeniable, rapid bactericidal efficacy against planktonic *C. acnes* in simplified *in vitro* settings, translating these parameters to complex *in vivo* microenvironments requires significant caution. First, surface sampling methods used in short-term clinical trials often fail to capture the persistence of *C. acnes* protected within robust, three-dimensional multi-species biofilms deep within the pilosebaceous unit (Byrd et al., 2018; Fournière et al., 2020); moreover, as with many microbiological models, susceptibility observed in mono-species laboratory assays cannot be directly translated to these complex cutaneous communities. Second, the physiological impact of highly acidic formulations depends heavily on concentration, vehicle, exposure time, and individual barrier integrity. As illustrated by Gerber et al. (2014), the application of high-concentration GA can precipitate severe adverse reactions, including painful erosions and scarring, particularly when the epidermal barrier is concomitantly compromised by factors such as low-dose isotretinoin therapy. This highlights a recurring translational challenge in dermatological research: the threshold at which AHAs compromise rather than bolster the epidermal shield is highly dependent on the experimental model and formulation parameters such as pH and free acid content (Tang and Yang, 2018). Reflecting safety concerns regarding unbuffered acids, European regulatory guidelines recommend limiting cosmetic GA formulations for home use to 4% at a pH of at least 3.8 (Almeman, 2024), a parameter clinically proven to maintain SC integrity without disrupting lipid bilayers (Fartasch et al., 1997). In stark contrast, specific *ex vivo* data reveal that significantly higher concentrations (e.g., 10%–25%), when properly buffered to a pH of approximately 4.0, promote epidermal renewal, stimulate collagen synthesis, and do not elevate pro-inflammatory TNF-α levels (Narda et al., 2021). Furthermore, clinical biophysical studies confirm that buffered acidic skin care products formulated around pH 4 safely maintain physiological skin pH without significantly impairing barrier integrity (Schulte To Brinke et al., 2021). Therefore, macroscopic toxicity observed in low-pH, unbuffered laboratory assays does not directly equate to clinical outcomes of buffered formulations.

Regardless of the formulation, aggressive exfoliation carries the risk of depleting the skin of its protective commensals. Clinical trials have demonstrated that high-concentration peels, such as 30% supramolecular salicylic acid, can perturb the biodiversity of the skin microbiota, reducing the populations of commensal *Staphylococcus* species (Shao et al., 2023). Importantly, while often described simply as microbial reduction, such sustained depletion of keystone taxa can disrupt ecological network resilience, potentially driving the system toward a true ecological dysbiosis rather than mere species suppression. Concurrently, AHA-induced chemical stress increases UV sensitivity (Kornhauser et al., 2010) and can provoke cutaneous irritation and erythema (Almeman, 2024). A severe clinical sequela of this compromised barrier integrity is the potential for these procedures to trigger dyschromia, secondary bacterial and fungal infections, scarring, or herpes simplex virus (HSV) reactivation (Nikalji et al., 2012; Gerber et al., 2014; Soleymani et al., 2018; Sarkar et al., 2025).

### 2.4 Lactic acid

Studies have demonstrated that certain AHAs may exhibit selective bactericidal properties that largely spare commensal bacteria (Green et al., 2009). A prime example of such an AHA is LA, which constitutes a vital metabolite of *S. epidermidis* (Salgaonkar et al., 2022). *In vitro* assays mimicking the acidic pH of these microbial ferments (pH 4.6) indicate that exposure to 0.5% LA has a minimal impact on the growth of this commensal microorganism while effectively inhibiting pathogens (Salgaonkar et al., 2022). Regarding its direct effects on the epidermis, topically applied creams containing LA at low concentrations (1.5%, 3%, and 5%) induce a concentration-dependent increase in the number of apoptotic cells in reconstructed human epidermis models (Rendl et al., 2001; Kornhauser et al., 2010). This modulated cellular turnover, accompanied by the release of vascular endothelial growth factor (VEGF) at lower concentrations, contributes to therapeutic effects, such as the treatment of photoaging (Rendl et al., 2001; Kornhauser et al., 2010). These regenerative properties parallel the effects of other AHAs, such as GA, which has been shown to accelerate *in vitro* cellular proliferation and *in vivo* collagen synthesis (Kim et al., 1998; Kornhauser et al., 2010). Furthermore, murine models demonstrate that GA exhibits photoprotective potential, significantly inhibiting UV-induced skin tumorigenesis (Kornhauser et al., 2010).

However, while murine models provide valuable mechanistic insights, they exhibit fundamental structural and microbiological differences compared to human skin. Notably, murine skin produces significantly lower quantities of triglyceride-rich sebum (Byrd et al., 2018). This physiological difference deprives lipophilic commensals of the optimal substrates needed to generate FFAs via lipases—a biochemical step that promotes bacterial adherence to the skin (Byrd et al., 2018). Consequently, murine models exhibit a distinct microbiome composition characterized by a lower abundance of these microorganisms (Byrd et al., 2018). A prime example is *C. acnes* (Sfriso et al., 2020), which typically dominates the sebaceous niches—such as the face, chest, and back—of the human ecosystem (Byrd et al., 2018). Therefore, extrapolating these effects directly to human clinical outcomes requires significant caution. Crucially, in contrast to GA, which elicits a rapid release of IL-1α from epidermal keratinocytes (Okano et al., 2003), LA does not elicit significant changes in the secretion of IL-8 (interleukin-8) (Rendl et al., 2001; Kornhauser et al., 2010). Conversely, in human reconstructed epidermis models, LA promotes the secretion of VEGF. However, this increase was noted exclusively with 1.5% and 3.0% LA formulations. At higher concentrations (5%), the results lacked statistical significance (Rendl et al., 2001). Concurrently, a concentration-dependent decrease in the secretion of angiogenin was observed (Rendl et al., 2001; Kornhauser et al., 2010). Ultimately, this specific, dose-dependent modulation of cytokine secretion by lactic acid is proposed to represent a mechanism contributing to its therapeutic effects, such as the treatment of photoaging (Rendl et al., 2001; Kornhauser et al., 2010).

## 3 Beta-hydroxy acids

### 3.1 Physicochemical characteristics of beta-hydroxy acids

BHAs, characterized by a beta-position hydroxyl group, are weak acids employed in topical dermatological therapies to manage acne vulgaris (AV) and improve cutaneous health (Rodan et al., 2016; Lu et al., 2019). Prevalent BHAs include beta-hydroxybutyric acid (BHB)—an endogenous ketone body prevalent in human metabolism (Qi et al., 2022; Yoshimura and Fujii, 2025) and salicylic acid, characterized by a pKa of 2.97 (Mamaligka and Dodou, 2024) and a molecular weight of 138.13 g/mol (Wiśniewska et al., 2023). Chemically, SA is an aromatic phenolic acid (2-hydroxybenzoic acid) (Favre and Powell, 2013), yet conventionally categorized as a BHA in dermatological literature (Arif, 2015). Due to SA’s poor aqueous solubility (1.97 g/L at pH 2.1) (Mamaligka and Dodou, 2024), professional formulations (20%-50%) are predominantly ethanol-based (Arif, 2015). However, it is crucial to note that SA forms crystals in low-pH alcoholic solutions, which cause skin irritation (Bilal et al., 2023). Moreover, its easy recrystallization in skin-irritating organic solvents can precipitate acute side effects such as prolonged erythema, stinging, and dryness (Zhou et al., 2023).

### 3.2 Mechanism of action and physiology of beta-hydroxy acids

**Figure 4:**
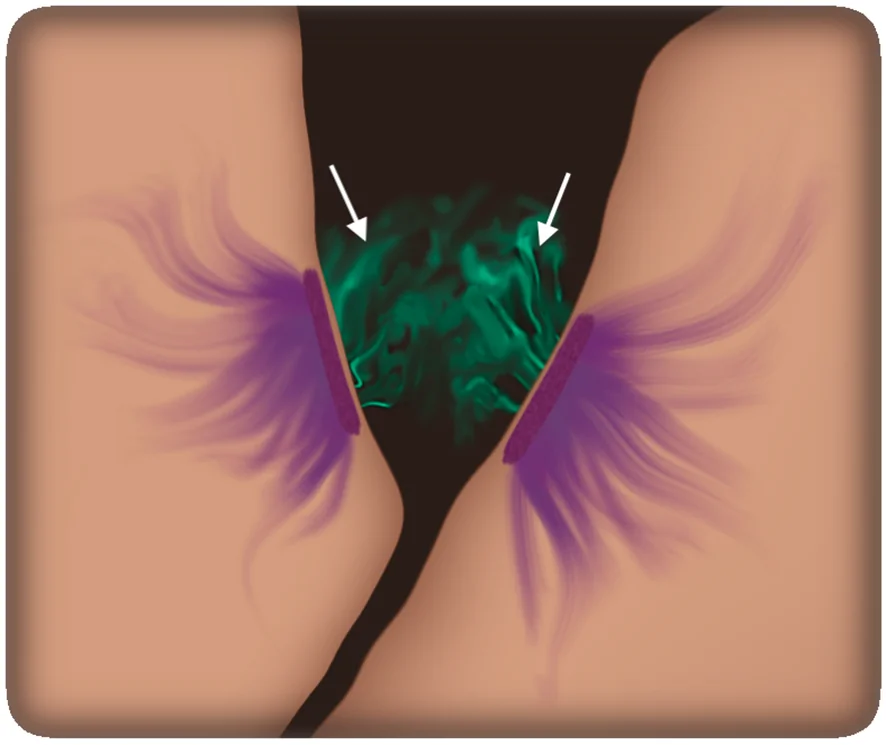
Potent desmolytic effect of salicylic acid (SA) on epidermal cellular adhesions. The schematic demonstrates profound structural disruption and dissolution of desmosomes, correlating with exfoliation and barrier modulation. White arrows highlight the dissolved desmosomal matrix. *SA*, salicylic acid.

BHA exfoliative mechanisms contrast with AHAs, due to their lipid-soluble nature (Arif, 2015). Furthermore, owing to its highly lipophilic profile, low pKa, and compact molecular structure, SA exhibits swift and deep permeation across the epidermal lipid barriers (Soleymani et al., 2018). SA formulations range from 0.5% to 50% concentrations (Arif, 2015; Lu et al., 2019). For optimal clinical efficacy, the pH of SA peels is maintained between 3.0 and 4.0, whereas in a simple aqueous solution, SA exhibits a pH of approximately 2.4 (Măgerusan et al., 2023). SA functions primarily as a desmolytic agent through a dual mechanism. It extracts desmosomal proteins, notably desmogleins, severing cellular connections to induce desquamation (**Figure 4**) (Arif, 2015). Concurrently, recent transcriptomic analyses demonstrate that SA upregulates the expression levels of serine proteases, specifically kallikrein-5 (*klk5*) (Zhou et al., 2023). Its profound lipophilicity dictates its follicular tropism, allowing it to easily permeate and infiltrate lipid-rich pilosebaceous layers and sebaceous glands (Arif, 2015; Al-Talib et al., 2017; Lu et al., 2019).

### 3.3 Salicylic acid

In contrast to AHAs—such as GA, which requires active alkaline neutralization to arrest keratocoagulation—SA is distinguished by its high lipophilicity and inherent self-neutralizing properties (Soleymani et al., 2018). While SA is generally considered to display a self-limiting action within the epidermis, the precise mechanism must be carefully distinguished: the characteristic white frost on the skin surface is merely the physical precipitation and crystallization of the acid (Arif, 2015), whereas the actual biochemical self-neutralization is mediated by the skin’s endogenous lipoproteins (Soleymani et al., 2018). However, this safety mechanism is not absolute. As emphasized by Soleymani et al. (2018), the depth of SA-induced tissue injury is highly cumulative; applying excessive amounts or consecutive layers of the solution can overwhelm the skin’s buffering capacity, driving unintended keratocoagulation deeper into the papillary dermis. Furthermore, extensive application carries a hidden risk of systemic toxicity, known as salicylism, which can occur even across intact epidermal barriers (Arif, 2015; Tabatabai et al., 2025). Nevertheless, under standard clinical protocols, SA restricts its activity predominantly to the lipid-rich pilosebaceous units (Arif, 2015; Lu et al., 2019; Măgerusan et al., 2023). Consequently, its impact on the cutaneous microbiome and local tissue architecture must be analyzed with particular reference to these specific anatomical niches.

#### 3.3.1 The impact of salicylic acid on the skin and microbiota

**Figure 5:**
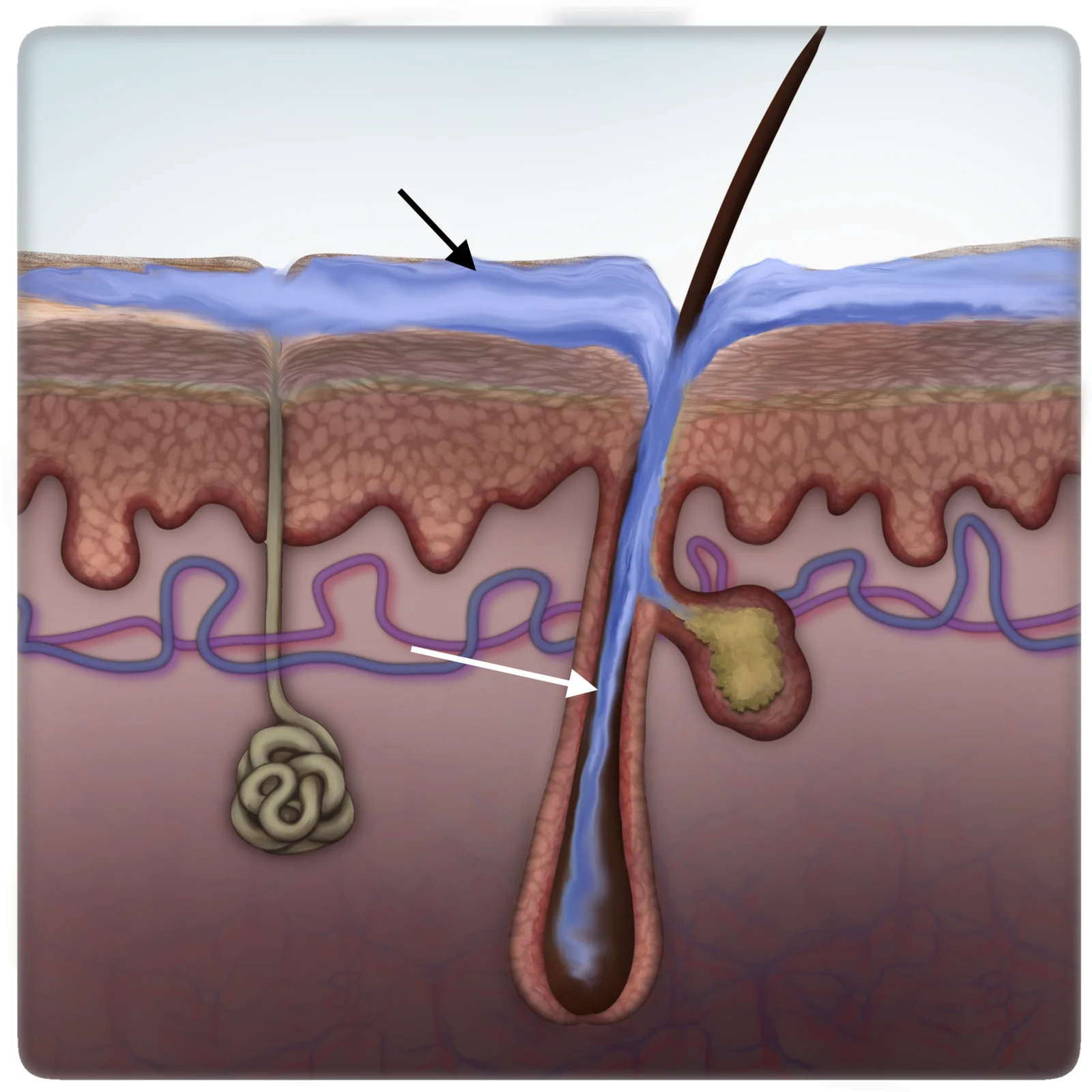
Cutaneous penetration profile of salicylic acid (SA). Due to its lipophilic nature, SA exhibits minimal diffusion through the moist stratum corneum. Instead, it effectively bypasses the superficial layers to penetrate deeply along the lipid-rich pilosebaceous unit (hair follicle and sebaceous gland). Black and white arrows trace the deep penetration pathway of the acid into the pilosebaceous unit. *SA*, salicylic acid.

Fundamentally, salicylic acid exhibits primarily desmolytic activity (Arif, 2015). However, at lower concentrations or with monolayer applications, its keratocoagulative effects do not penetrate into the deeper layers of the skin (Soleymani et al., 2018). Its impact is observed predominantly within the SC and the pilosebaceous units, the ecological niches occupied by bacteria of the genera *Staphylococcus* and *Cutibacterium* (Grice and Segre, 2011; Fournière et al., 2020; Byrd et al., 2018), respectively. Owing to its excellent lipophilicity, SA penetrates selectively and easily along the pilosebaceous units (**Figure 5**) (Arif, 2015; Al-Talib et al., 2017; Soleymani et al., 2018). In the *in vitro* SEB-1 cell line model, it has been demonstrated to attenuate sebocyte lipogenesis via the downregulation of the AMP-activated protein kinase (AMPK)/SREBP-1 pathway (Lu et al., 2019). Furthermore, when inflammation was experimentally induced in these cells using heat-inactivated *C. acnes*, SA reduced the inflammatory response (Lu et al., 2019). Importantly, these molecular mechanisms appear to translate into clinical efficacy; *in vivo* perilesional skin biopsies from patients treated with 30% supramolecular salicylic acid (SSA) peels revealed a significant downregulation of pro-inflammatory markers, corroborating its anti-inflammatory potential in a real-world setting (Shao et al., 2023).

The activation of AMPK by salicylic acid transmits a signal that inhibits SREBPs. These proteins serve as the primary transcription factors for genes regulating the production of cholesterol and fatty acids (Elias, 2005), which are essential for sebum synthesis (Lu et al., 2019). Consequently, within this specific *in vitro* model, SA action resulted in a 40% reduction in the mRNA transcript levels of both SREBP-1a and SREBP-1c in sebocytes. The decline in SREBP-1 transcription factors leads to the inhibition of fatty acid synthase (FAS) and acetyl-CoA carboxylase (ACC), the pivotal enzymes for sebum production. In this manner, SA was shown to downregulate the gene expression levels of FAS by 28.1% and ACC by 33.4% (Lu et al., 2019). Importantly, these antilipogenic mechanisms appear to translate into clinical efficacy; *in vivo* measurements from patients treated with 30% SSA peels revealed a significant reduction in facial sebum levels, corroborating its sebosuppressive potential in a real-world setting (Shao et al., 2023).

At this juncture, it is critical to emphasize that sebum constitutes a vital nutritional source for the cutaneous microbiota (Grice and Segre, 2011; Byrd et al., 2018; Swaney et al., 2023). Human skin presents a dry, cool, mildly acidic, and nutrient-poor environment (Scharschmidt and Segre, 2025). A significant proportion of commensal bacteria are lipophilic microorganisms for which sebum components, such as triglycerides, squalene, and wax esters, serve as an indispensable food source for survival (Garlet et al., 2024; Tao et al., 2024). This phenomenon is termed lipid auxotrophy, and representatives of microorganisms deriving their nutrition in this manner include the fungi *Malassezia* and certain bacteria of the genus *Corynebacterium* (Byrd et al., 2018; Vijaya Chandra et al., 2021; Swaney et al., 2023). A prime example of an auxotrophic bacterium is *Corynebacterium kefirresidentii*, which, much like many other commensals, has an obligate requirement for the presence of sebum, as it lacks the fatty acid synthase I (FAS I) enzyme necessary to synthesize fatty acids required for cell envelope construction. *C. acnes*, as demonstrated in studies, resides within the anaerobic environment of sebaceous glands, and the population density of these microorganisms increases in direct proportion to the volume of sebum secreted (Byrd et al., 2018; Wei et al., 2022). Adequate sebum production is also essential due to its innate antimicrobial properties (Grice and Segre, 2011). Free fatty acids (FFAs) can be toxic to numerous potentially pathogenic bacteria (Ushijima et al., 1984; Chen et al., 2011; Wojciechowska and Dos Santos Szewczyk, 2025). The degradation of sebum by commensal bacteria induces a substantial reduction in the pH on the skin surface, which guards against colonization by pathogens such as *S*. *aureus* and *Streptococcus pyogenes* (Grice and Segre, 2011; Choi and Kang, 2024; Lambers et al., 2006). Pathogenic bacteria, including *S. aureus* and *S. pyogenes*, thrive optimally in a neutral pH environment (approx. 7.0) (Elias, 2005; Hülpüsch et al., 2024). This neutral pH stimulates the virulence of *S. aureus* because, under these conditions, the *agr* (accessory gene regulator) system—responsible for toxin expression within the quorum sensing mechanism (Jordan et al., 2022; Tamai et al., 2023)—exhibits peak activity (Hülpüsch et al., 2024). Commensal microorganisms, however, have evolved survival mechanisms in the presence of sebum. Over 80% of *S*. *epidermidis* strains possess the capacity to esterify FFAs to cholesterol, thereby neutralizing their bactericidal effects (Swaney et al., 2023). When introduced into this delicate lipid and microbial ecosystem, salicylic acid (SA) exerts profound modulatory effects. Importantly, *in vivo* studies have observed a downregulated expression of pro-inflammatory markers—IL-1α, IL-6 (interleukin-6), and IL-17 (interleukin-17), as well as transforming growth factor beta (TGF-β) and toll-like receptor 2 (TLR-2)—in patients treated with SA (Shao et al., 2023), alongside a stabilization of microbial dysbiosis and reduction of clinical acne lesions (Bilal et al., 2023). These clinical observations are fundamentally supported by diverse methodological approaches. Specifically, SA-mediated suppression of intracellular inflammatory pathways has been confirmed both *in vitro* in human sebocytes and *in vivo* using a rabbit ear acne model (Lu et al., 2019). Furthermore, a complex modulation of *S. aureus* virulence has been observed in separate *in vitro* assays (Dotto et al., 2021). Notably, *in vitro* studies indicate that while SA suppresses the *S. aureus agr* quorum-sensing system—thereby downregulating the production of acute extracellular toxins—this exact mechanism paradoxically stabilizes its biofilms, theoretically increasing the risk of chronic bacterial persistence (Dotto et al., 2021). However, as previously emphasized, extrapolating these mechanistic findings directly to clinical outcomes requires significant caution. Recent research highlights a profound nonconformity in *S. aureus* biofilm formation between *in vitro* assays and *in vivo* models, demonstrating that *agr* inhibition does not actually enhance biofilm development in living organisms (Jordan et al., 2022). Therefore, the concern regarding SA-induced biofilm stabilization may be of limited clinical relevance. This methodological discrepancy compounds the previously discussed ecological limitations of laboratory and animal models. This constitutes a profound contrast to the action of GA and LA, which represent the AHAs. As previously mentioned, *in vitro* models have shown that GA triggers the release of IL-1α by keratinocytes (Okano et al., 2003), whereas LA promotes the secretion of VEGF (Kornhauser et al., 2010; Rodan et al., 2016).

#### 3.3.2 Cytotoxic and bactericidal activity

**Figure 6:**
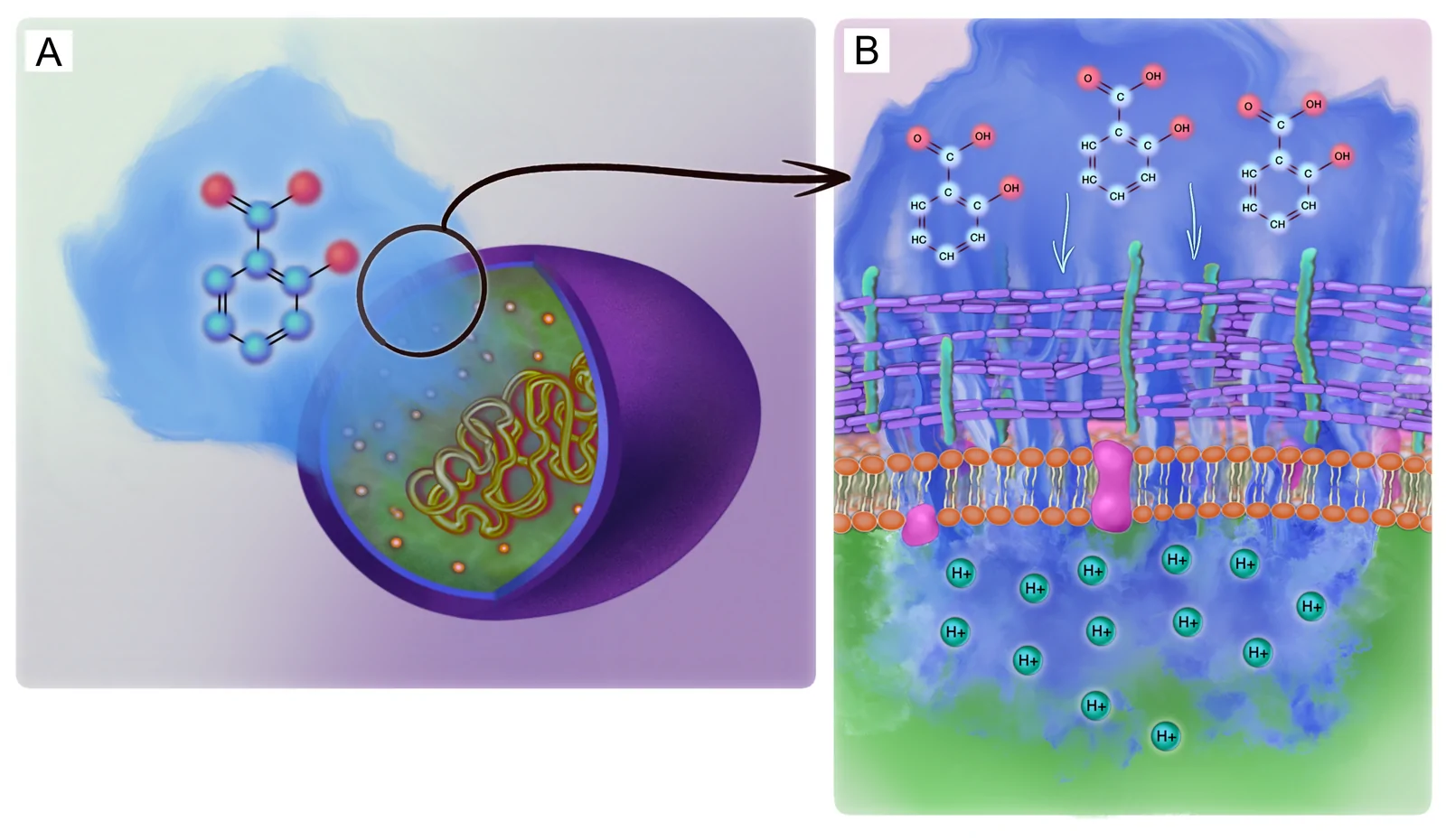
Conceptual model based on current literature illustrating the mechanism of salicylic acid (SA)-induced intracellular acidification. **(A)** Schematic representation of lipophilic SA molecules approaching and penetrating the bacterial cell envelope. **(B)** The black arrow pointing from panel **(A, B)** highlights a detailed cross-section of the bacterial cell wall and lipid bilayer; passive diffusion of SA leads to rapid intracellular acidification by an influx of protons (H^+^). Light blue arrows indicate the disruption of cellular homeostasis. *SA*, salicylic acid; *H^+^*, hydrogen proton.

It must be noted that bacterial cells, encompassing both pathogenic and commensal organisms, possess lipid membranes that are highly permeable to lipophilic compounds (Chen et al., 2011; Arif, 2015; Suriyaprom et al., 2022). The bacterial microbiota of the human face largely comprises Gram-positive organisms characterized by cell walls composed of a thick peptidoglycan layer, which does not constitute a barrier to lipophilic agents (Grice et al., 2009; Fournière et al., 2020; Lee et al., 2021). Salicylic acid applied to the mildly acidic skin surface exists predominantly in its ionized, negatively charged state; however, the remaining undissociated, uncharged fraction possesses a high capacity for passive diffusion across bacterial lipid envelopes (Ricke, 2003; Suriyaprom et al., 2022; Laudouze et al., 2025). Upon entering the bacterial cytoplasm, where the pH is near neutral, the SA molecule undergoes rapid dissociation, releasing protons and anions (Ricke, 2003). The liberated protons drastically lower the cytoplasmic pH, inducing intracellular acidification (Cotter and Hill, 2003; Ricke, 2003; Laudouze et al., 2025). This leads to the impairment of intracellular structures, including the denaturation of proteins and DNA, disruption of enzymatic homeostasis, and depletion of cellular ATP reserves consumed by active proton-extruding ATPases attempting to maintain pH homeostasis, ultimately resulting in growth arrest and cell death (Cotter and Hill, 2003; Ricke, 2003; Suriyaprom et al., 2022). Acidification of the extracellular environment potentiates the bactericidal efficacy of SA due to a shift in the dissociation equilibrium toward the protonated form, which readily penetrates bacterial membranes (Suriyaprom et al., 2022; Laudouze et al., 2025). This phenomenon, driven by the transmembrane pH gradient and conceptually illustrated in **Figures 6A, B**, has been demonstrated *in vitro* to results in an intracellular accumulation of SA at concentrations 10 to 30 times higher than those outside the cell in *E. coli* models (Creamer et al., 2017). However, as *E. coli* is a Gram-negative gut resident, the structural and physiological complexities of extrapolating these *in vitro* findings to the human skin microbiota—which is predominantly composed of Gram-positive organisms—remain highly challenging. Therefore, future studies are warranted to evaluate these intracellular accumulation kinetics directly within representative members of the cutaneous microbiome.

Therefore, considering its potentially lethal consequences for numerous commensal bacteria, high-concentration SA therapy should be administered judiciously and primarily in cases of confirmed seborrhea (Arif, 2015; SanMiguel et al., 2017; Soleymani et al., 2018; Lu et al., 2019; Bilal et al., 2023). Hyperseborrhea promotes the pathological proliferation and dominance of virulent *C. acnes* strains, particularly phylotype IA1, which are implicated in the pathogenesis of AV (Shao et al., 2023; Zhang et al., 2024; Scharschmidt and Segre, 2025; Pakosiński et al., 2026). Fungi of the genus *Malassezia* possess the capacity to degrade sebum triglycerides via potent lipases, resulting in the release of irritating oleic acid, thereby contributing to pityriasis versicolor and seborrheic dermatitis (Ushijima et al., 1984; Grice et al., 2009; Vijaya Chandra et al., 2021; Carmona-Cruz et al., 2022; Tao et al., 2024). However, the utilization of SA without clear dermatological indications may precipitate facial skin dysbiosis and epidermal barrier impairment (SanMiguel et al., 2017; Blicharz et al., 2021; Callejon et al., 2023; Wojciechowska and Dos Santos Szewczyk, 2025). Consequently, this facilitates pathogenic colonization, potentially leading to complications with an *S. aureus* or *S. pyogenes* etiology (Jinnestål et al., 2014; Paller et al., 2019; Edslev et al., 2021; Sarkar et al., 2025). This stems from the fact that aggressive chemical exfoliation may significantly reduce the natural microbiota, which serves as the skin’s frontline defense against pathogen invasion. This process can severely compromise the skin’s innate defense mechanisms conferred by the commensal flora, including the deliberate acidification of the microenvironment resulting from fermentation into short-chain fatty acids (SCFAs), direct competition for niches and nutrients, and the production and release of pathogen-inhibiting bacteriocins (Wang et al., 2014a, Wang et al., 2014b; Fournière et al., 2020; Chen et al., 2021; Salgaonkar et al., 2022; Zheng et al., 2022; Tamai et al., 2023; Hülpüsch et al., 2024).

### 3.4 Beta-hydroxybutyric acid

In contrast to salicylic acid, BHB is structurally a true representative of the β-hydroxy acid group, according to strict chemical nomenclature (Qi et al., 2022). The topical application of BHB to facial skin is a relatively novel therapeutic modality that still warrants extensive investigation. Nevertheless, initial reports highlighting its beneficial cutaneous effects are emerging (Qi et al., 2022; Yoshimura and Fujii, 2025). In the human body, BHB is synthesized predominantly as a hepatic metabolite during periods of fasting or rigorous physical exertion, functioning primarily as a ketone body (Qi et al., 2022). Studies investigating its systemic effects have established that BHB acts as an endogenous inhibitor of class I histone deacetylases (HDACs) (Shimazu et al., 2013; Qi et al., 2022). Class I HDACs are enzymes responsible for modulating the degree of DNA condensation; their inhibition, coupled with direct protein modification via lysine β-hydroxybutyrylation, results in the regulation of gene expression, notably those encoding NF-κB, TP53, and MYC (Qi et al., 2022). Furthermore, BHB possesses the capacity to suppress the NLRP3 inflammasome pathway by inhibiting the activation and subsequent assembly of the NLRP3 multiprotein complex (Youm et al., 2015). This inflammasome is integral to initiating inflammatory cascades across numerous pathological states, thereby positioning BHB as a potentially potent anti-inflammatory agent (Qi et al., 2022). BHB attenuates inflammasome formation through several multifaceted cellular mechanisms. By interacting with ion channels located in the plasma membrane, BHB impedes potassium ion efflux, maintaining a high physiological intracellular concentration of K^+^ that serves as an inhibitory signal for NLRP3 inflammasome activation (Youm et al., 2015; Qi et al., 2022). Moreover, this compound promotes the activation of the AMPK signaling pathway via HCAR2 (GPR109A) receptors, which has been shown to alleviate endoplasmic reticulum (ER) stress and, crucially for dermatological applications, preserve epidermal barrier integrity (Qi et al., 2022; Yoshimura and Fujii, 2025). As an endogenous molecule, it also functions as a direct ligand for specific G protein-coupled receptors (GPCRs) residing on the cell surface, including the HCAR2/GPR109A free fatty acid receptors (FFARs) (Qi et al., 2022). The activation of the HCAR2 receptor induces the suppression of the AC/cAMP/PKA signaling cascade, thereby blocking the activation of the NF-κB transcription factor and attenuating its downstream pathway (Qi et al., 2022). The inhibition of this factor downregulates the expression of pro-inflammatory genes, which consequently manifests as a diminished release of pro-inflammatory cytokines (Qi et al., 2022). Research has also validated the impact of BHB on mitochondrial function. Through oxidative processes, BHB has the capacity to facilitate the closure of mitochondrial permeability transition pores (mPTPs), which is crucial for maintaining the electrochemical gradient required for ATP synthesis. Additionally, this mechanism safeguards cells against apoptosis induced by an excess of ROS (Shimazu et al., 2013; Qi et al., 2022). Importantly, recent investigations (2025) have progressively refined the proposed anti-allergic mechanisms of BHB in contact hypersensitivity, correcting prior assumptions of direct immune cell modulation. While initial *in vivo* findings hypothesized that systemically elevated BHB might directly activate GPR109A/HCAR2 receptors on skin mast cells to suppress IgE-mediated degranulation (Yoshimura et al., 2025), subsequent cellular and immunofluorescence analyses have disproved direct mast cell targeting (Yoshimura and Fujii, 2025). Specifically, HCAR2 expression in cutaneous tissue is localized predominantly within the epidermal keratinocyte layers and does not colocalize with mast cells (Yoshimura and Fujii, 2025). Consequently, the anti-allergic efficacy of both systemic and topical BHB relies on an indirect, barrier-protective pathway mediated by the HCAR2–CaMKK–AMPK signaling cascade in keratinocytes (Yoshimura and Fujii, 2025). Activation of this epidermal pathway preserves the continuous localization of tight junction proteins Zo-1 and occludin, reinforcing skin barrier integrity and preventing allergen penetration, which subsequently blocks downstream mast cell activation and degranulation at the challenge site (Yoshimura and Fujii, 2025). Similar to salicylic acid, the positive impact of BHB on the skin is linked to the activation of the AMPK pathway. However, the fundamental distinction in the mechanism of action between these two acids is that SA activates this enzyme within sebocytes—ultimately culminating in the suppression of sebum production (Lu et al., 2019)—whereas, in the case of BHB, the cascade (the HCAR2-CaMKK-AMPK axis) is initiated within epidermal keratinocytes (Yoshimura and Fujii, 2025). Moreover, topically applied BHB exhibits a protective role by preserving the structural integrity of proteins composing tight junctions, specifically occludin and ZO-1 proteins. This action reinforces the epidermal barrier, impeding the penetration of pathogens and allergens deep into the cutaneous strata. The localized application of BHB in models of allergic contact dermatitis (ACD) of the ear drastically diminished edema and inhibited mast cell degranulation. This effect appears to be mediated primarily via action on keratinocytes, as mast cells are devoid of HCAR2 receptors. The prevention of their degranulation is thus an indirect consequence of epidermal barrier reinforcement and the preclusion of pathogen and allergen ingress (Yoshimura and Fujii, 2025). In the context of antimicrobial activity, BHB is well-characterized regarding the gut microbiota, where it exerts a bacteriostatic effect (Qi et al., 2022). It inhibits the proliferation of bacterial genera such as *Bifidobacterium* and *Lactobacillus*, as well as the majority of intestinal anaerobes, concurrently reducing α-diversity (Qi et al., 2022). Conversely, there is currently a conspicuous lack of research focusing directly on the impact of BHB on the facial skin microbiota. Extrapolating these gastrointestinal findings to the cutaneous ecosystem is highly speculative and ecologically flawed. The gut represents an anaerobic, nutrient-rich niche where BHB suppresses specific anaerobes like *Bifidobacterium* (Qi et al., 2022); this starkly contrasts with the aerobic, lipid-driven microenvironment of the facial SC, which selectively harbors lipophilic and halotolerant flora such as *Cutibacterium* and *Staphylococcus* species (Scharschmidt and Segre, 2025). Therefore, whether BHB exerts a similar diversity-reducing effect on cutaneous flora remains a profound knowledge gap.

## 4 Polyhydroxy acids

### 4.1 Physicochemical characteristics of polyhydroxy acids

PHAs represent a class of chemical compounds characterized by the presence of a hydroxyl group in the alpha position relative to the carboxyl group, coupled with multiple hydroxyl groups throughout the molecular structure. Among the compounds within this group utilized in dermatological practice, lactobionic acid (LBA) and gluconolactone are prominent (Green et al., 2009; Măgerusan et al., 2023). Due to the presence of numerous hydroxyl groups, PHAs possess significantly higher molecular weights compared to compounds from the AHA or BHA classes. This multiplicity of hydroxyl groups confers highly hydrophilic properties and a substantial capacity for water retention (Coroli et al., 2021; Piątek-Gołda et al., 2025). Lactobionic acid (LBA) is a direct derivative of lactose, generated through the selective oxidation of the glucose moiety to gluconic acid (Coroli et al., 2021; Sáez‐Orviz et al., 2022). In terms of acidity, LBA is comparable to GA, with a pKa ranging from 3.6 to 3.8 (Green et al., 2009; Hachem et al., 2010). As dictated by its molecular architecture, it exhibits exceedingly poor solubility in organic solvents (Piątek-Gołda et al., 2025).

### 4.2 Mechanism of action and physiology of polyhydroxy acids

**Figure 7:**
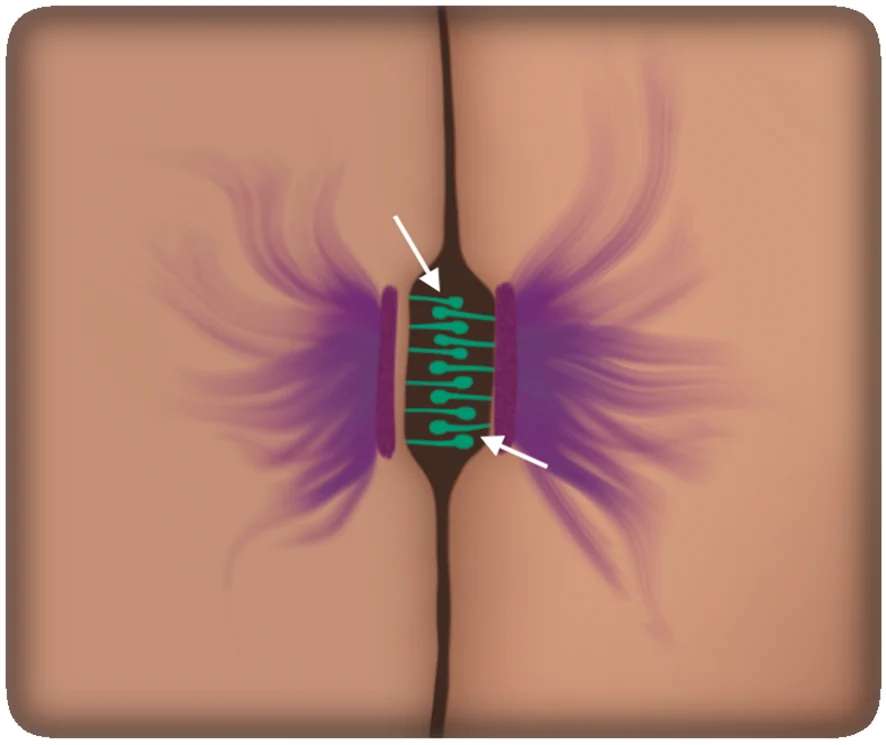
Restorative effect of lactobionic acid (LBA) on epidermal cellular adhesions. In stark contrast to classical hydroxy acids, LBA application promotes the structural cohesion and strengthening of desmosomes, thereby reinforcing the integrity of the epidermal barrier. White arrows point to the reinforced, highly cohesive desmosomal structures. *LBA*, lactobionic acid.

Among PHAs, the most prominent representatives are LBA, classified as a bionic acid, and gluconolactone (Green et al., 2009; Măgerusan et al., 2023). These acids act predominantly in a synergistic and multifaceted manner (Piątek-Gołda et al., 2025). Both compounds exert superficial effects and induce mild exfoliation. They have been shown to positively modulate the activity of hydrolytic enzymes involved in ceramide synthesis, which translates to the maintenance of optimal skin hydration and the preservation of barrier function (Hachem et al., 2010; Choi and Kang, 2024). Studies have also demonstrated their inhibitory effect on serine proteases, which attenuates the premature degradation of corneodesmosomes and supports the structural integrity of the epidermis (**Figure 7**) (Hachem et al., 2010). Furthermore, LBA has been identified as an inhibitor of matrix metalloproteinases (MMPs), thereby helping to mitigate collagen degradation and potentially delaying the subsequent formation of rhytides induced by intrinsic aging and UV radiation (Green et al., 2009; Coroli et al., 2021). Both acids also exhibit intrinsic antioxidant properties due to their capacity to chelate metal ions, particularly Fe^2+^ ions, thereby reducing the generation of deleterious reactive oxygen species (ROS) (Green et al., 2009).

### 4.3 Lactobionic acid

#### 4.3.1 The impact of LBA on facial skin

LBA exfoliation is superficial and significantly milder than AHAs or BHAs, safely avoiding immediate visible desquamation even in sensitive skin profiles (Green et al., 2009; Măgerusan et al., 2023). This gentle action stems from its high molecular weight, extreme hydrophilicity, and potent humectant properties, which robustly promote water retention (Green et al., 2009; Coroli et al., 2021; Piątek-Gołda et al., 2025). Rather than penetrating deeply, LBA permeates the entire non-viable SC, artificially and sustainably lowering the pH across all strata, as demonstrated in both murine models and human *in vivo* studies utilizing 10% LBA solutions (Hachem et al., 2010; Tasić‐Kostov et al., 2019). Its exfoliative mechanism relies on indirect enzymatic modulation via compartment acidification (Hachem et al., 2010). Within the deeper layers of the SC, LBA selectively lowers the pH of extracellular membrane microdomains, which regulates lipid maturation processes and epidermal cohesion (Hachem et al., 2010).

Maintaining a low SC pH upregulates hydrolytic enzymes critical for ceramide synthesis, specifically β-glucocerebrosidase and acid sphingomyelinase (Elias, 2005; Hachem et al., 2010; Choi and Kang, 2024). Concurrently, this acidification inhibits neutral/alkaline-preferring serine proteases (kallikrein-related peptidases, KLKs) within the lower SC strata, attenuating premature desquamation (Elias, 2005; Deraison et al., 2007; Hachem et al., 2010; Blicharz et al., 2021). In murine models, this acidification has been shown to preserve desmoglein-1 (DSG1) in the upper SC while inducing desmoglein-3 (DSG3) expression and concurrently decreasing DSG1 in deeper epidermal layers (Hachem et al., 2010). Furthermore, KLK enzymes exist as inactive complexes bound to the LEKTI protein, alongside pro-KLK5 and pro-KLK7 (kallikrein-7) proenzymes (Deraison et al., 2007; Hachem et al., 2010). While reduced pH provokes KLK-LEKTI dissociation, profound microdomain hyperacidification substantially suppresses their proteolytic capacity, and proenzymes become sequestered within lipid rafts, which restricts premature maturation (Deraison et al., 2007; Hachem et al., 2010). Conversely, on the cutaneous surface, KLK-LEKTI dissociation occurs without lipid raft sequestration or such hyperacidification, allowing KLKs—particularly KLK5—to retain residual proteolytic activity for controlled desquamation (Deraison et al., 2007; Hachem et al., 2010).

#### 4.3.2 Antioxidant, chelating, and occlusive properties of LBA

It is crucial to note that LBA possesses intrinsic antioxidant properties and the capacity to chelate transition metal ions (Coroli et al., 2021; Sáez‐Orviz et al., 2022; Piątek-Gołda et al., 2025). Consequently, KLKs are not the sole enzymes inhibited by LBA. Based on its chemical structure, it is hypothesized that LBA, as a large organic anion, has the capacity to bind zinc ions (Zn^2+^) (Upadhya and Strasberg, 2000; Hong et al., 2025). This proposed mechanism is thought to underlie its activity as an inhibitor of MMPs (Green et al., 2009; Hong et al., 2025), whereas its capacity to chelate iron ions (Fe^2+^) has been shown *in vitro* to suppress the generation of free hydroxyl radicals (Green et al., 2009). In the skin, MMPs are responsible for the degradation and destruction of extracellular matrix components, encompassing collagenases and gelatinases (Upadhya and Strasberg, 2000; Hong et al., 2025). Furthermore, the chelation of metal ions, particularly Fe^2+^, may restrict the formation of highly reactive free hydroxyl radicals, as these ions serve as catalysts in oxidative propagation (Green et al., 2009). In the context of topical LBA application to facial skin, this mechanism is hypothesized to offer photoprotective benefits, particularly since ultraviolet (UV) radiation induces oxidative stress and generates ROS within the skin (Green et al., 2009). Hence, the presence of LBA is proposed to help mitigate UV-induced ROS propagation, potentially protecting against structural defects and photoaging (Green et al., 2009). LBA—much like the previously discussed GA—has been shown to sequester calcium ions (Fartasch et al., 1997; Upadhya and Strasberg, 2000; Hong et al., 2025). While both acids are hypothesized to share this calcium-chelating mechanism to facilitate the loosening of corneodesmosomes (Fartasch et al., 1997; Hong et al., 2025), LBA’s significantly larger molecular size causes it to penetrate the skin more slowly and act more superficially. This is thought to allow for gentle desmosomal degradation with a lower risk of the irritation frequently associated with GA (Green et al., 2009; Sáez‐Orviz et al., 2022; Piątek-Gołda et al., 2025). Crucially, this shared ability to chelate calcium is hypothesized to influence bacterial aggregation and biofilm stability in laboratory models. Because extracellular DNA (eDNA) relies heavily on electrostatic forces and calcium ions to mediate bacterial aggregation and stabilize mature biofilms *in vitro* (Das et al., 2014), the sequestration of Ca^2+^ by LBA is suggested to disrupt the structural integrity of the biofilm matrix. While this calcium-chelating action is hypothesized to participate in epidermal desmosomal degradation, LBA has also been shown *in vitro* to inhibit biofilm formation and reduce the expression of virulence-associated factors in pathogens such as *S. aureus*.

**Figure 8:**
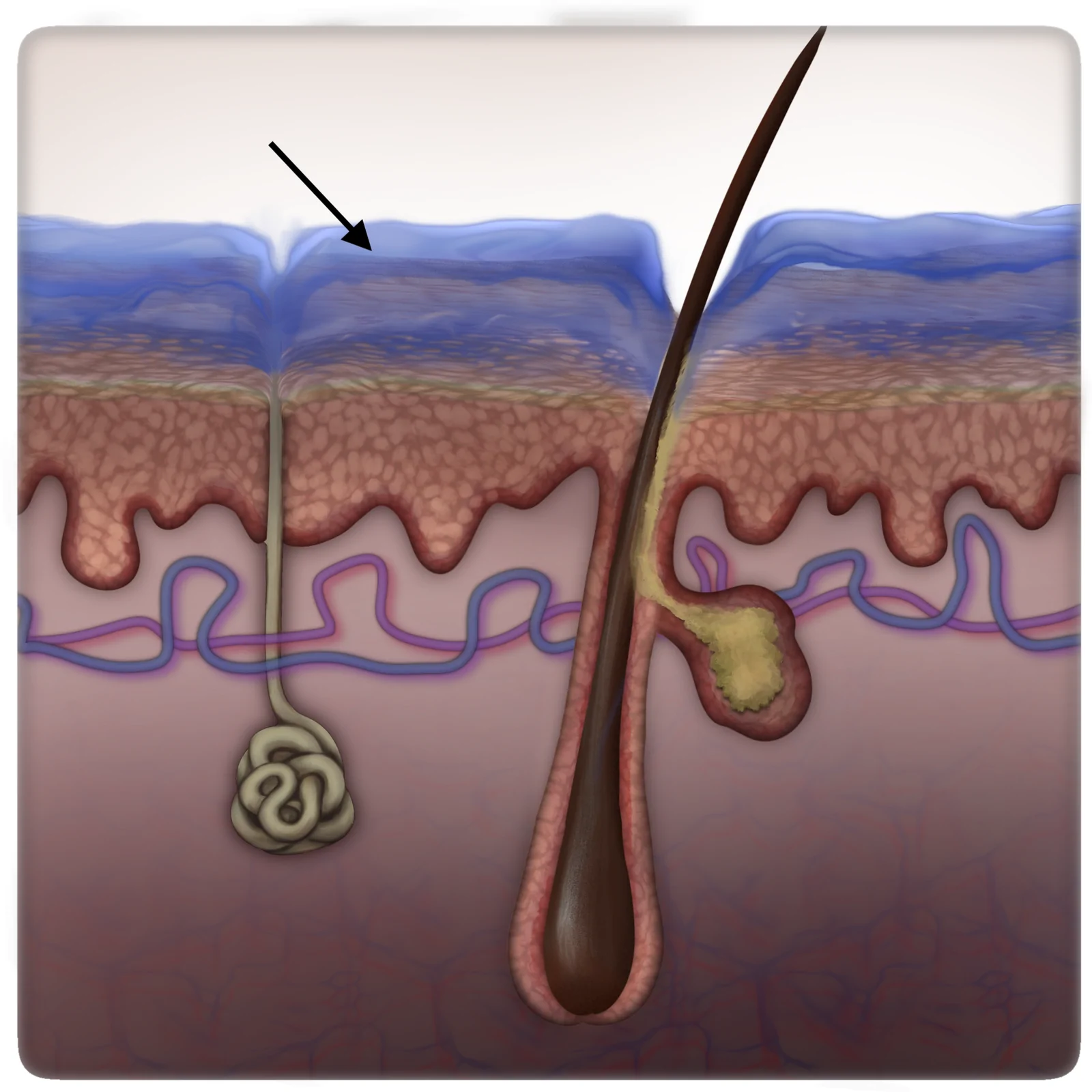
Cutaneous penetration profile of lactobionic acid (LBA). The thick surface overlay indicates that LBA, a large polyhydroxy acid molecule, forms a substantial, moisture-binding protective mantle on the epidermal surface, with virtually no deep tissue penetration. Arrows indicate the large lactobionic acid molecules remaining strictly on the outer epidermal surface. *LBA*, lactobionic acid.

Furthermore, it has been demonstrated that the evaporation of aqueous LBA solutions at room temperature results in the formation of a distinct physical mantle (**Figure 8**) (Green et al., 2009). Due to the exceptionally hygroscopic properties of LBA, governed by the presence of numerous hydroxyl groups, this matrix retains and incorporates approximately 14% water within its structure (Green et al., 2009; Coroli et al., 2021; Piątek-Gołda et al., 2025). When applied topically (e.g., in a prevalent 10% concentration), this property is hypothesized to allow LBA to form a physical mantle on the skin surface (Green et al., 2009; Hachem et al., 2010). This mantle is suggested to exert an occlusive and soothing effect on the skin, potentially acting as a supplementary barrier that protects the epidermis from desiccation and supports moisturization, without causing barrier impairment or pathological increases in transepidermal water loss (TEWL) (Green et al., 2009; Tasić‐Kostov et al., 2019; Măgerusan et al., 2023). Ultimately, due to LBA’s cohesive effects in the deeper SC, its exfoliative action is thought to occur predominantly within the most superficial layer—the SD (Fartasch et al., 1997; Hachem et al., 2010). The action of LBA is proposed to rely on restoring and stabilizing the skin’s pH to a value approximating the physiological norm or inducing beneficial hyperacidification (Hachem et al., 2010; Schulte To Brinke et al., 2021). In murine models, this acidification has been shown to optimize key enzymatic cascades and improve lipid processing, rather than relying solely on the direct disruption of corneodesmosomal bonds—a mechanism more typical of smaller AHAs (Fartasch et al., 1997; Hachem et al., 2010; Măgerusan et al., 2023). Consequently, this mechanism is hypothesized to enhance the physiological function of the epidermal barrier. Furthermore, because the physical and chemical features of the skin—particularly its acidic nature—select for adapted microbial communities, restoring this physiological pH is considered essential for maintaining a balanced skin microbiome (Grice and Segre, 2011). By accelerating barrier recovery in disrupted skin, LBA helps normalize cutaneous permeability without causing pathological increases in TEWL frequently associated with irritation (Hachem et al., 2010; Tasić‐Kostov et al., 2019).

#### 4.3.3 The impact of LBA on facial skin microbiota

**Figure 9:**
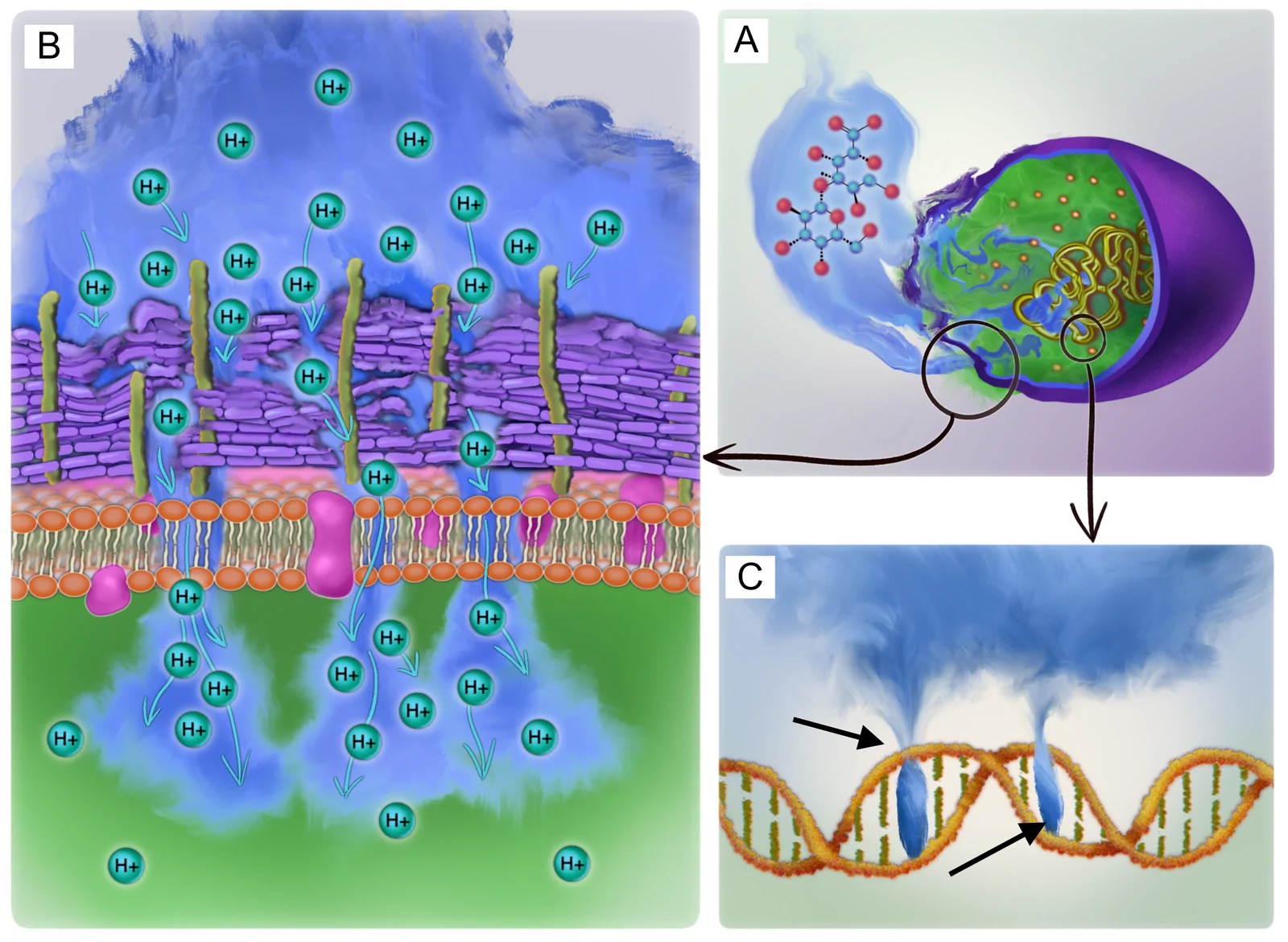
Conceptual model based on current literature illustrating structural envelope disruption and hypothesized DNA intercalation induced by lactobionic acid (LBA). **(A)** The black arrow pointing from panel **(A, B)** shows the structural collapse of the bacterial cell mediated by LBA. Light blue arrows show passive proton influx. **(B)** Severe degradation of the cell wall and membrane integrity. **(C)** The arrow pointing from **(B, C)** denotes the proposed model of potential intercalation. Within this panel, the black arrow highlights LBA moieties into the bacterial double helix, which is hypothesized to disrupt DNA replication and transcription *in vitro*.Abbreviations: *LBA*, lactobionic acid; *DNA*, deoxyribonucleic acid; *H^+^*, hydrogen proton.

Beyond the aforementioned beneficial cutaneous effects, LBA has been shown to exhibit antimicrobial properties against specific bacterial isolates *in vitro* (Kang et al., 2020; Coroli et al., 2021). However, while these laboratory findings indicate a capacity to affect individual bacteria, extrapolating this *in vitro* bactericidal or bacteriostatic activity directly to the modulation of the complex cutaneous microflora *in vivo* requires caution. Studies utilizing mono-species *in vitro* models have demonstrated its antibacterial activity against *S. aureus* and other Gram-positive bacteria, proposing the disruption of the cell wall as a primary mechanism of action (Cao et al., 2019; Kang et al., 2020; Sáez‐Orviz et al., 2022). Under these specific laboratory conditions, LBA is observed to induce structural damage to the cell envelope, leading to profound morphological deformations and the loss of cell wall boundary integrity (**Figures 9A, B**). This acid-induced disruption is reported to facilitate the leakage of intracellular components, such as alkaline phosphatase (AKP), from the periplasmic space into the extracellular environment. Imaging obtained via transmission electron microscopy (TEM), scanning electron microscopy (SEM), and confocal laser scanning microscopy (CLSM) has further revealed irregular cellular morphologies, numerous deformations, complete lysis, and the disruption of mono-species biofilms (Cao et al., 2019; Kang et al., 2020; Hou et al., 2022; Kang et al., 2024). Furthermore, based on molecular docking and *in vitro* spectroscopic assays, it has been hypothesized that LBA might interact with genomic DNA via intercalation (**Figure 9C**); however, this remains a proposed theoretical model that has not yet been experimentally verified *in vivo* within complex cutaneous ecosystems (Kang et al., 2020; Sáez‐Orviz et al., 2022).

Furthermore, based on *in vitro* mono-species models, LBA is reported to disrupt bacterial biofilms by inhibiting the release of extracellular polysaccharides (EPS) and eDNA (Kang et al., 2024). This anti-biofilm activity is also hypothesized to involve the upregulation of *GlpQ*, an enzyme suggested to degrade wall teichoic acid (WTA) within the bacterial cell envelope, thereby reducing early-stage adhesion and aggregation (Hou et al., 2022). Research elucidating the impact of LBA on *S. aureus* has also revealed that this acid strongly suppresses hemolytic activity, primarily through the downregulation of *hla* gene expression (Kang et al., 2024). Efficacy against MRSA (methicillin-resistant *Staphylococcus aureus*) has also been corroborated; differences in MIC (Minimum Inhibitory Concentration) values between strains are hypothesized to be influenced by variations in their biofilm-producing abilities rather than solely their resistance profiles (Kang et al., 2024). For methicillin-susceptible strains, the MIC in a 2019 study by Cao et al. was reported as 15 mg/mL, whereas in a more recent 2024 study by Kang et al., this value was 8 mg/mL for the *S. aureus* ATCC 25923 strain (Cao et al., 2019; Kang et al., 2024). Conversely, for the more resilient MRSA, the MIC in an older 2020 study by Kang et al. was 18.75 mg/mL, and in a 2022 study by Hou et al., it was 12.5 mg/mL for the N315 and SJTU21564 isolates. The authors in both cases utilized the MRSA N315 strain, while Hou et al. additionally investigated the SJTUF21564 isolate (Kang et al., 2020; Hou et al., 2022). As noted by researchers, discrepancies in MIC values for identical strains may stem from methodological divergences—such as agar well diffusion versus broth microdilution—and variations in the purity of the LBA solutions procured from different manufacturers (Cao et al., 2019; Hou et al., 2022; Kang et al., 2024). The Minimum Bactericidal Concentration (MBC) was also determined, which for standard *S. aureus* was reported as 50 mg/mL (Cao et al., 2019; Kang et al., 2020). Crucially, while these parameters define specific antimicrobial activity *in vitro*, the concentrations most frequently utilized in topical formulations are 10%, which corresponds to 100 mg/mL (Cao et al., 2019; Tasić‐Kostov et al., 2019). Although this concentration significantly exceeds established MICs, extrapolating these *in vitro* bactericidal effects directly to the modulation of the complex cutaneous microflora *in vivo* requires caution. Research indicates that LBA at a concentration of approximately 0.7 mg/mL exerts a bacteriostatic effect against the indicator commensal *S. epidermidis in vitro*, which the bacteria can overcome within 24 hours; however, it is reported that concentrations exceeding this threshold exert bactericidal activity against various Gram-positive and Gram-negative bacteria in laboratory settings (Coroli et al., 2021). While the 100 mg/mL (10%) concentration present in commercially available preparations significantly exceeds these *in vitro* thresholds, extrapolating this difference to suggest a definitive risk of commensal eradication *in vivo* requires caution. The complex environment of the skin microbiome may modulate these effects, though the potential impact on *S. epidermidis* populations warrants consideration. Currently, comprehensive studies determining the impact of LBA on other commensal microorganisms, such as *Corynebacterium* spp. and *Malassezia* spp., are lacking; nevertheless, given that *Corynebacterium* spp. belong to the Gram-positive group, a potential susceptibility of their populations to LBA could be hypothesized based on *in vitro* observations (Fournière et al., 2020; Sáez‐Orviz et al., 2022). The literature also currently lacks investigations into the effects of LBA on microorganisms implicated in the pathogenesis of common skin diseases affecting sebum-rich areas. The hydrophilic nature of LBA is hypothesized to restrict its penetration into the pilosebaceous units—which are colonized by potentially pathogenic *C. acnes* and *Malassezia* spp.—during standard topical application (Coroli et al., 2021; Piątek-Gołda et al., 2025). Considering its reported antimicrobial activity, however, determining the *in vitro* MIC and MBC for these organisms, particularly the acne-dominant phylotypes such as *C. acnes* phylotype IA1, could represent a critical step toward developing novel therapeutics. Demonstrating the *in vitro* efficacy of LBA against these microorganisms would pave the way for the application of advanced carrier systems, hypothetically enabling this compound to reach the aforementioned structures and target these specific pathogens. LBA has also been shown *in vitro* to exhibit antibacterial activity against Gram-negative bacteria, akin to its action on Gram-positive species (Kang et al., 2020; Sáez‐Orviz et al., 2022). For instance, it is suggested that this acid primarily targets the cell envelope, rapidly increasing its permeability in a dose-dependent manner and potentially inducing hypoosmotic shock (Sáez‐Orviz et al., 2022). Electron microscopy imaging of *P. fluorescens* has visualized structural damage under laboratory conditions, encompassing the loss of cell wall boundaries, irregular wrinkling, and pore formation (Kang et al., 2020). LBA is reported to induce cytoplasmic leakage from Gram-negative bacterial cells, ultimately leading to a profound loss of cellular viability *in vitro* (Kang et al., 2020; Sáez‐Orviz et al., 2022). Similar to observations in Gram-positive bacteria, it has been suggested that LBA could potentially interact with genomic DNA via intercalation under specific *in vitro* conditions; the physiological relevance of this pathway *in vivo* remains a significant knowledge gap (Kang et al., 2020; Sáez‐Orviz et al., 2022). Furthermore, the potential inhibition of folate biosynthesis—which blocks DNA and protein synthesis—and the reduction of intracellular ATP concentrations have been noted in the literature (Sáez‐Orviz et al., 2022). Crucially, these specific antibacterial mechanisms were investigated primarily on microorganisms that do not represent typical facial skin pathogens: *Pseudomonas fluorescens*, *Vibrio parahaemolyticus*, and *Salmonella typhimurium* (Kang et al., 2020; Sáez‐Orviz et al., 2022). Although the facial skin is significantly dominated by Gram-positive microorganisms, the commensal species inhabiting this niche also include Gram-negative organisms crucial for maintaining cutaneous homeostasis (Byrd et al., 2018; Chen et al., 2021). These include *Roseomonas mucosa* and bacteria of the *Acinetobacter* genus (Romano-Bertrand et al., 2016; Zhang et al., 2024). While these microbes constitute a minor fraction of all organisms colonizing the facial skin, studies have demonstrated their potential health-promoting effects. For instance, bacteria of the *Acinetobacter* genus are suggested to stimulate immune system cells to produce IL-10 and induce a Th1 immune response, potentially protecting against allergic sensitization and inflammation (Fyhrquist et al., 2014; Zhang et al., 2024). *R. mucosa* has also been hypothesized to mitigate UV-induced damage via the secretion of specific exopolysaccharides that alleviate inflammation, decrease epidermal cell apoptosis, and reduce pro-inflammatory cytokine levels (Zhan et al., 2025). Research on rosacea has reported a depletion in the populations of *Acinetobacter* spp. and *R. mucosa* (Zhang et al., 2024). While studies determining the direct impact of LBA on these organisms are lacking, their classification as Gram-negative bacteria suggests a potential susceptibility to LBA *in vitro.* However, extrapolating these laboratory findings to firmly deduce their eradication *in vivo* upon topical application of LBA requires extreme caution, as the complex skin microenvironment may significantly modulate these effects. Therefore, it can be postulated that their viability may be significantly compromised.

### 4.4 Gluconolactone

Gluconolactone (GDL), alongside LBA, represents one of the primary representatives of the PHAs utilized in dermatology and topical therapeutics (Green et al., 2009). From a chemical standpoint, GDL is a PHA derived from glucose (Măgerusan et al., 2023). Its substantial molecular size is reported to restrict deep and rapid epidermal penetration, causing it to penetrate the skin more slowly than smaller AHAs (Măgerusan et al., 2023; Piątek-Gołda et al., 2025). This slower penetration mechanism is suggested to render GDL highly tolerable, minimizing the risk of irritation often associated with abrupt changes in epidermal pH (Green et al., 2009; Măgerusan et al., 2023). Concurrently, this superficial action facilitates the manifestation of skin-beneficial properties characteristic of PHAs, including the sustained acidification of the SC, gentle exfoliation, and antioxidant and potential photoprotective activities (Green et al., 2009; Hachem et al., 2010; Măgerusan et al., 2023). However, despite these elegant biophysical, clinical, and host-oriented therapeutic insights, a rigorous microbiological characterization of GDL remains strikingly absent in the literature. While its capacity to reduce stratum corneum pH and biochemically accelerate permeability barrier recovery is well-demonstrated *in vivo* (Hachem et al., 2010), there is a complete scarcity of studies investigating its direct antimicrobial or antibiofilm efficacy. To date, no established MICs, MBCs, or time-kill kinetics have been reported for pure GDL against key cutaneous pathobionts such as *S. aureus* or *C. acnes* (Fournière et al., 2020; Măgerusan et al., 2023). Instead, direct microbiological data in acne therapy are often derived from its hydrolyzed form, gluconic acid, which has been evaluated primarily as a botanical polysaccharide complex (such as MPA-Regul™) (Fournière et al., 2020). Although this complex has been shown *in vitro* to mitigate the cellular virulence of aggressive *C. acnes* phylotypes (RT4 and RT5) without inducing cytotoxicity, and has clinically contributed to reducing acne lesions (Fournière et al., 2020), pure GDL itself lacks rigorous, isolated validation. Furthermore, no clinical trials utilizing high-throughput sequencing have evaluated GDL’s impact on the taxonomic composition or the α- and β-diversity of the human skin microbiota. Consequently, while GDL is highly promising as a barrier-preserving and theoretically microbiome-sparing acidifying agent, validating its precise ecological footprint on the living skin microbiome remains a critical, yet entirely vacant, priority for future dermatological research.

## 5 Discussion

### 5.1 Comparative mechanistic overview of hydroxy acids

To synthesize the mechanistic divergence of these chemical exfoliants, it is essential to contrast their distinct biochemical profiles and the resulting ecological impact on the skin (**Table 2**). GA (AHA), characterized by its minimal molecular weight, penetrates deeply into the epidermis, triggering rapid corneodesmosome degradation and TRPV1-dependent keratinocyte proliferation (Denda et al., 2010; Kornhauser et al., 2010). However, this non-specific acidification frequently elicits a pro-inflammatory response, notably the release of IL-1α, which drives a transient pro-inflammatory cascade necessary for dermal matrix remodeling but requires strict physiological control (Okano et al., 2003). In stark contrast, salicylic acid (BHA) leverages its lipophilicity to selectively target sebum-rich pilosebaceous units. Beyond mere exfoliation, SA functions as a potent metabolic modulator, suppressing sebocyte lipogenesis via the AMPK/SREBP-1 pathway and mitigating inflammation, while *in vitro* evidence suggests it suppresses the *agr* quorum-sensing system, which reduces certain extracellular virulence factors but paradoxically stabilizes *S. aureus* biofilms (Lu et al., 2019; Dotto et al., 2021; Shao et al., 2023). Meanwhile, LBA (PHA) represents a paradigm shift toward barrier-protective exfoliation. Its substantial molecular weight restricts its action to the SC, where its potent chelating properties simultaneously disrupt bacterial biofilm matrices via calcium binding and alleviate oxidative stress through iron chelation (Green et al., 2009; Coroli et al., 2021; Kang et al., 2024). Unlike the acute pH fluctuations induced by AHAs, LBA promotes sustained, gentle acidification that optimally recalibrates the KLK-LEKTI proteolytic balance and upregulates ceramide-synthesizing enzymes (Hachem et al., 2010). Ultimately, ecological models confirm that pH modulation is the primary stimulus for dysbiosis (Greugny et al., 2022; Hülpüsch et al., 2024; Choi and Kang, 2024), reducing diversity and facilitating colonization by opportunistic species, which promotes inflammatory cascades.

### 5.2 Translating *In vitro* antimicrobial efficacy to *in vivo* skin ecology

When evaluating the antimicrobial properties of hydroxy acids, translating strict *in vitro* MIC and MBC parameters to clinical dermatological efficacy requires significant caution. Standard microbiological assays evaluate free-floating (planktonic) bacteria in nutrient-rich media at neutral pH under continuous 24-hour exposure. This experimental paradigm stands in sharp contrast to the ecological reality of human skin, where the cutaneous microbiota predominantly organizes into complex, three-dimensional biofilms (Blicharz et al., 2021). Bacteria encapsulated within a mature EPS matrix exhibit profoundly heightened resistance, often requiring antimicrobial concentrations orders of magnitude higher than standard planktonic MIC values to achieve effective eradication. Furthermore, the temporal dynamics of topical treatments—such as rapid vehicle evaporation or the short contact times typical of rinse-off chemical peels—render standard 24-hour *in vitro* incubation unrepresentative from a clinical perspective (Callejon et al., 2023). Nevertheless, emerging empirical evidence indicates that dermatological interventions, including chemical peels, actively modulate the microbiome by altering the cutaneous microenvironment *in vivo* (Ellis et al., 2024). Standard cosmetic regimens have been shown to alter microbial diversity and abundance, demonstrating that even non-antimicrobial skincare routines reshape the skin’s ecological balance (Hwang et al., 2021; Wagner et al., 2024). Recent *in vivo* studies demonstrate that the regular application of low-pH formulations (pH < 5) consistently enhances the diversity of the natural skin microbiome and can reduce the relative abundance of opportunistic pathobionts, such as *Corynebacterium* (Janssens-Böcker et al., 2025).

### 5.3 Remaining knowledge gaps and future perspectives

While summarizing the mechanistic impacts of AHAs, it is vital to outline future research directions and acknowledge current limitations. For instance, while eATP is traditionally viewed merely as a host regenerative and inflammatory signal (Denda et al., 2010), it has been hypothesized that it could also participate in complex, inter-kingdom purinergic cross-talk. *In vitro* studies have demonstrated that eATP gradients can serve as a signaling mechanism that alters the twitching motility and organization of *Pseudomonas aeruginosa* biofilms (Nolan et al., 2015). Consequently, it is biologically plausible to postulate that the massive GA-induced efflux of eATP might act as an environmental cue modulating the behavior of cutaneous microbiota or disrupting pathogenic biofilms. However, it must be strongly emphasized that such eATP-driven microbiome modulation remains a proposed hypothesis. To date, this mechanism is inferred by bridging independent *in vitro* observations and has not yet been directly demonstrated experimentally within the complex ecological environment of the human skin microbiome. Validating these inter-kingdom signaling pathways and their actual impact on skin dysbiosis represents a critical priority for future *in vivo* studies. In addition to the aforementioned purinergic cross-talk, another compelling hypothesis for future investigation involves the structural destabilization of bacterial biofilms by AHAs. It is well-established that eDNA heavily relies on electrostatic forces and divalent cations, specifically calcium, to mediate bacterial aggregation and stabilize the mature biofilm matrix (Das et al., 2014; Kang et al., 2024). Concurrently, a fundamental mechanism of AHA-induced exfoliation relies on calcium ion chelation (Wang, 1999; Kornhauser et al., 2010). Consequently, it is biologically highly plausible to hypothesize that by sequestering these essential calcium ions, GA and other AHAs may exhibit potential antibiofilm properties through the destabilization of eDNA. However, it is critical to distinguish this proposed structural impairment from overall microbiome modulation. As biofilm destabilization can occur without measurable shifts in community structure or ecological function, further *in vivo* studies are required to establish whether this calcium-sequestering mechanism translates into the preservation of eubiosis within the SC and pilosebaceous units—key ecological niches (Grice and Segre, 2011; SanMiguel et al., 2017; Fournière et al., 2020). The depletion of commensal microbiota following aggressive chemical peeling provides a critical avenue for future research. While high-concentration salicylic acid peels are known to reduce *Staphylococcus* species (Shao et al., 2023), the downstream microbiological consequences of this reduction remain to be fully elucidated. Given that *S. epidermidis* is fundamentally required for generating protective ceramides and maintaining barrier homeostasis (Zheng et al., 2022), we hypothesize that its prolonged eradication by chemical exfoliants could precipitate an increase in TEWL. Because such physically impaired states heighten susceptibility to cutaneous colonization by pathogenic organisms like *S. aureus* (Jinnestål et al., 2014; Edslev et al., 2021), future longitudinal clinical studies should investigate whether hydroxy acid-induced dysbiosis directly drives these secondary infections.

While primary *in vitro* studies (Rendl et al., 2001), as well as subsequent reviews (Kornhauser et al., 2010), established that LA modulates epidermal cytokine profiles by increasing VEGF secretion at lower concentrations while concurrently decreasing angiogenin, the precise physiological translation of this balance warrants further discussion. Given the fundamental pro-angiogenic nature of VEGF, it is plausible to hypothesize that its upregulation promotes beneficial microcirculation, thereby improving tissue nourishment crucial for the regeneration of photodamaged skin (Rendl et al., 2001). Furthermore, the simultaneous concentration-dependent decrease in angiogenin may serve as a critical regulatory mechanism. In photoaged skin, chronic sun exposure frequently manifests as visible telangiectasia (Green et al., 2009), which is indicative of aberrant, chaotic vessel growth. Therefore, we hypothesize that the dual action of lactic acid—selectively stimulating VEGF while suppressing angiogenin—might represent a highly orchestrated modulation that enhances functional microvascular networks while actively mitigating the risk of chaotic angiogenesis.

Literature regarding the holistic impact of hydroxy acids on facial microbiota remains sparse; existing studies focus primarily on targeted pathogen eradication (*C. acnes, S. aureus*), largely overlooking the risk of broader chronic dysbiosis (SanMiguel et al., 2017). Next-generation acids (LBA, BHB) offer promising alternatives, although their precise impact on human skin commensals *in vivo* remains largely uncharacterized (Coroli et al., 2021; Kang et al., 2024). While baseline MIC and MBC/MFC data must be expanded for both commensals and pathobionts, future investigations must imperatively incorporate biofilm models and microbiome-sparing formulations. Evaluating the targeted antimicrobial activity of next-generation acids is of paramount importance given the escalating global crisis of antimicrobial resistance (Podwojniak et al., 2025). Rigorous, well-controlled clinical trials are required to refine clinical guidelines, ensuring that future dermatological and cosmetic strategies balance aesthetic outcomes with the long-term preservation of cutaneous microbial ecology (Wojciechowska and Dos Santos Szewczyk, 2025).

Despite the well-documented biophysical benefits of gluconolactone (GDL) in reducing skin surface pH, accelerating permeability barrier recovery, and demonstrating high cutaneous compatibility *in vivo* (Green et al., 2009; Hachem et al., 2010), a profound methodological gap persists regarding its direct microbiological characterization. In comprehensive reviews evaluating organic acids in dermatology, standard microbiological parameters—such as MICs, MBCs, or direct biofilm disruption assays—are notably absent for pure GDL (Fournière et al., 2020; Măgerusan et al., 2023). Instead, available microbiological data are primarily derived from its hydrolyzed form, gluconic acid, which has been evaluated only as a component of multi-component botanical polysaccharide complexes (such as MPA-Regul™) (Fournière et al., 2020). Although this complex has been shown *in vitro* to exhibit no induced cytotoxicity towards keratinocytes and no activation of virulence in aggressive *C. acnes* phylotypes (RT4 and RT5), pure GDL itself lacks isolated microbiological validation (Fournière et al., 2020). Furthermore, within the reviewed clinical literature, trials utilizing high-throughput sequencing to evaluate the impact of pure GDL on taxonomic composition or the α- and β-diversity of the human skin microbiota are absent (Fournière et al., 2020; Măgerusan et al., 2023). Similarly, the utilization of lactobionic acid (LBA) is constrained by industrial, regulatory, and conceptual limitations. The production of LBA remains highly polarized, with chemical synthesis offering high yields but relying on metallic catalysts that pose environmental risks, while sustainable biological methods are constrained by poor scalability and high costs (Piątek-Gołda et al., 2025). Moreover, while LBA displays *in vitro* antibacterial activity, inducing cell membrane damage and intracellular leakage in *S. aureus* (Cao et al., 2019), these findings primarily originate from food-preservation models and foodborne pathogens. Extrapolating such food-safety parameters to the complex, living skin microenvironment is highly problematic, especially since LBA’s preservative properties are heavily suppressed in complex, protein- or lipid-rich matrices due to macromolecular masking (Sáez‐Orviz et al., 2022). Furthermore, a total lack of human *in vivo* trials establishing clinical safety and tolerance thresholds has caused a regulatory paralysis, leaving LBA without a harmonized European regulatory framework (such as EFSA) for widespread cosmetic and nutritional use (Sáez‐Orviz et al., 2022; Piątek-Gołda et al., 2025). Beyond individual acid profiles, broader methodological challenges restrict current cutaneous microbiome and skincare research. A fundamental causality dilemma persists in dermatology, as clinical trials remain correlative, failing to establish whether taxonomic dysbiosis is the precise cause or consequence of skin pathologies (Fournière et al., 2020; Edslev et al., 2021). Although high-throughput genomic profiling has fundamentally shifted the pathophysiological paradigm of acne vulgaris—demonstrating that the disease is triggered not by the simple proliferation of *C. acnes*, but by a distinct loss of intra-species phylotype diversity and the subsequent dominance of the highly pro-inflammatory phylotype IA1—establishing how topical hydroxy acids selectively modulate these delicate intra-species populations *in vivo* remains an unresolved challenge. Current literature often simplistically equates *in vitro* growth inhibition (MIC/MBC) against isolated planktonic cells with actual skin microbiome modulation, ignoring the extreme therapeutic resilience of mature, multi-species biofilms nested within lipophilic, anaerobic follicular niches *in vivo* (Fournière et al., 2020). Furthermore, dysbiosis must not be defined merely as the numerical depletion or proliferation of selected taxa, but as a complex ecological shift affecting community dynamics, functional redundancy, and host-microbe immunological crosstalk (Fournière et al., 2020; Edslev et al., 2021). To date, longitudinal clinical trials tracking the long-term ecological consequences of repeated, chronic low-pH exposure on cutaneous taxonomic resilience, functional stability, and barrier homeostasis remain completely absent. In terms of biofilm biophysics, structural models remain heavily biased toward eDNA and its stabilization via ionic bridging by divalent calcium (Ca^2+^) ions, which thermodynamically favors *P. aeruginosa* aggregation (Das et al., 2014). While the thermodynamic role of Ca^2+^ in cross-linking eDNA is established (Das et al., 2014), the correlation between such divalent cation dynamics and other cell-wall components—such as wall teichoic acids (WTA), whose enzymatic degradation by *GlpQ* is promoted during LBA-mediated *S. aureus* biofilm inhibition (Hou et al., 2022)—remains to be fully integrated in the context of cutaneous interfaces. To bypass these off-target ecological disruptions and barrier irritation, recent research has explored next-generation delivery systems, such as biodegradable zein-based films plasticized with oleic acid (Coroli et al., 2021). Although these protein-based films achieve controlled release of LBA and exert bacteriostatic activity against *E. coli* and *S. epidermidis in vitro* (Coroli et al., 2021), their translation to multi-species *in vivo* cutaneous ecosystems and their long-term impact on the host-microbe equilibrium have yet to be investigated.
